# Engineering Light-Responsive Transcription Factors via Strategic Masking of Post-translational Modification Residues

**DOI:** 10.1021/acs.bioconjchem.5c00561

**Published:** 2026-01-27

**Authors:** Raj V. Nithun, Shada Khoury, Muhammad Jbara

**Affiliations:** School of Chemistry, Raymond and Beverly Sackler Faculty of Exact Sciences, 26745 Tel Aviv University, Tel Aviv 69978, Israel

## Abstract

The development of synthetic transcription factors (TFs) that generate functional outputs in response to specific stimuli holds significant promise for modulating key cellular processes in both basic research and biomedical applications. Here, we rationally designed synthetic TFs bearing reversible modifications that mimic post-translational modifications regulatory mechanisms. By combining native chemical ligation (NCL) with palladium-mediated C–S cross-coupling, we synthesized a caged Max variant in which key residues (e.g., Lys31/57) were masked with o-nitroveratryloxycarbonyl groups. While the preparation of photoreactive proteins is generally incompatible with traditional NCL–desulfurization approaches, our strategy highlights the power of integrating total synthesis with late-stage transformations to access novel photoreactive proteins. Remarkably, whereas the engineered caged Max displayed a pronounced reduction in DNA-binding activity, potent binding to the enhancer box was rapidly restored upon site-selective unmasking of Lys31/57. The caged Max can be efficiently activated on-demand within minutes by simple in situ photolysis, enabling precise modulation of its DNA-binding activity. Our approach provides an effective means for producing and activating TF proteins, paving the way for light-responsive TF analogs with on-demand control across diverse applications.

## Graphical abstract

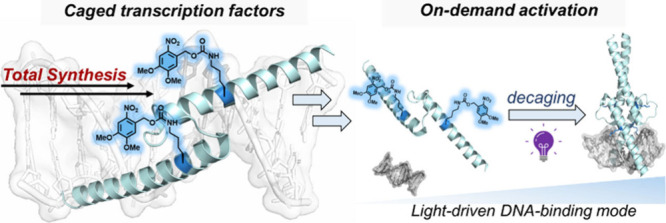

## Introduction

Gene expression is a fundamental biological process through which information encoded in genes is translated into functional products, such as RNA and proteins. Transcription factors (TFs) regulate this process at the transcriptional level by binding to specific DNA sequences within the enhancer or promoter region.^1^ Many TFs remain inactive in the cell until activated by specific stimuli, upon which they regulate gene expression.^2^ These stimuli can include interactions with binding partners that trigger conformational changes, ligands, environmental factors, or post-translational modifications (PTMs).^3^ The resulting responses frequently include conformational rearrangements and changes in binding affinities to interacting partners and/or DNA, which in turn alter gene recognition and transcriptional output. Engineering TF systems that can selectively bind to target DNA sequences in response to external stimulation have significant potential for fundamental research and therapeutic applications.^4^ Although potential activation mechanisms have been studied in peptides, extending this level of control to proteins remains challenging and scarce due to the difficulty of generating site-specifically caged TFs using conventional biological methods.^5^

Among the hundreds of TFs encoded in the human genome, Max plays a central role in regulating cell growth, proliferation, and differentiation.^6^ It controls the expression of ∼15% of human genes through partner-selection mechanisms as part of the Myc–Max–Mad TF system.^7^ For instance, Myc/Max heterodimers recognize the enhancer box (E-box) sequence to activate gene expression, whereas Max/Max homodimers can compete for the same E-box sites to repress transcription. Disruption of this regulatory network is implicated in nearly 70% of human cancers.^8^ Interestingly, Max has been found to undergo phosphorylation and acetylation, which have been shown to modulate its DNA binding activity and nuclear localization.^9^ We envisioned that developing synthetic TFs bearing reversible transformations, mimicking the regulatory mechanisms of PTMs, would enable on-demand TF activation in response to external stimuli and provide a powerful tool for various applications. In principle, the design of caged TFs with masked key residues for DNA interactions provides a promising strategy to activate DNA binding activity through site-selective TF decaging mechanisms.

The engineering of synthetic photoreactive TFs provides a powerful strategy to modulate TF–DNA interactions with spatiotemporal precision via light activation. Our design draws on the well-established capacity of PTMs to modulate TF structure and function.^10^ For example, Lys acetylation can alter Max–DNA interactions by neutralizing the ε-amino group, thereby interfering with interactions with the DNA backbone. We envision incorporating acetylation mimics at key Lys sites using a photoreactive group that undergoes facile and selective cleavage upon irradiation, which can potentially modulate TF–DNA interactions through a rapid unmasking strategy (Figure 1). In this study, we developed a light-responsive caged TF system derived from Max that can be unmasked and activated by rapid UV irradiation. Using one-pot native chemical ligation (NCL), combined with late-stage S-arylation, we synthesized caged Max in which key Lys residues (K31 and K57) were masked with the o-nitroveratryloxycarbonyl (Nvoc) group. DNA binding studies showed that potent DNA binding activity was restored following photolysis. These findings pave the way for designing light-responsive systems for the temporal control of TF activity.

**Figure 1:**
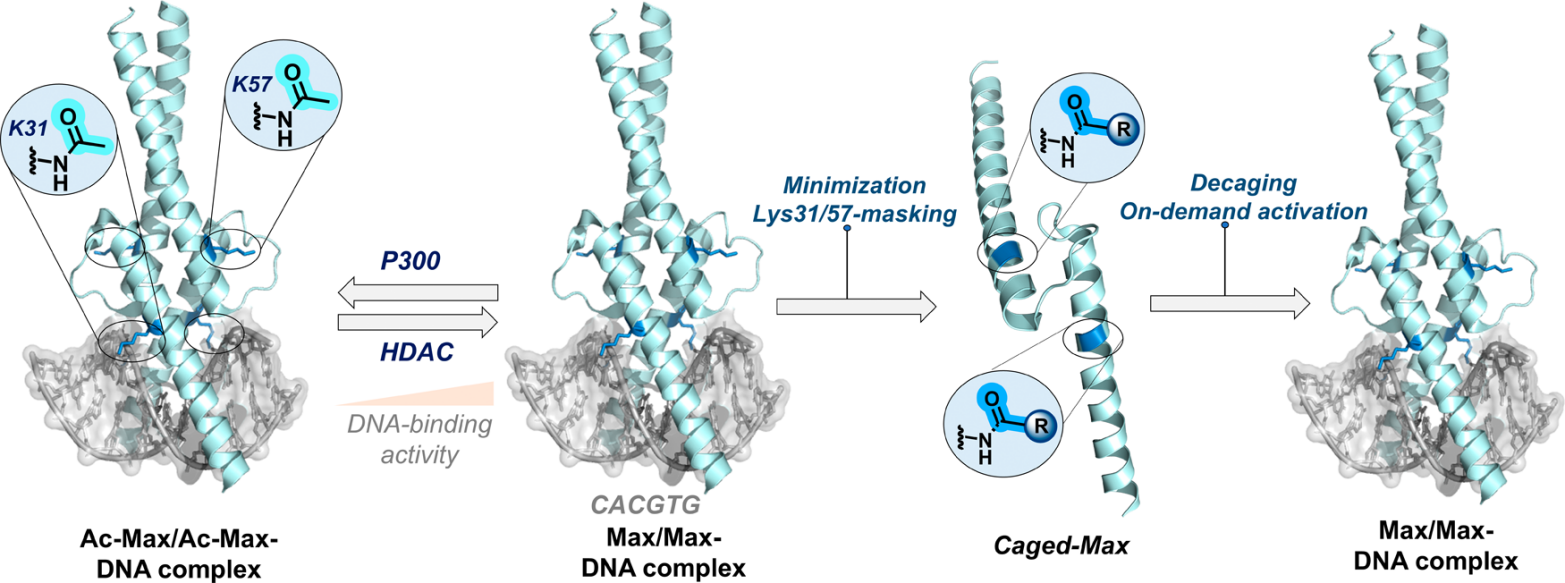
Representation of the designed stimuli-responsive caged transcription factors for on-demand activation and DNA binding. The caged proteins were derived from post-translationally modified Max; (R-caging group) (PDB: 1HLO).

## Results and Discussion

In our strategy for designing stimuli-responsive TFs, the initial step was to identify the key residues essential for DNA contact. We have recently reported the total chemical synthesis of Max TF, which allowed us to elucidate the molecular role of Ser-phosphorylation and Lys-acetylation at key residues within the DNA binding domain.^11^ Importantly, our findings revealed that acetylation at Lys31 and Lys57 significantly inhibits DNA binding activity by disrupting critical salt bridges between the ammonium residue and the phosphate diester backbone (Figure 1). By probing the role of the Lys31 and Lys57 residues in the DNA binding activity of Max, we have recently engineered abiotic TFs by replacing these residues with noncanonical residues, which led to the discovery of advanced Max modulators (μMax) bearing Lys to L-homoarginine (hArg) mutations.^12^ Remarkably, the developed μMax analogs exhibited potent DNA binding activity, remarkable cell permeability, and effectively suppressed Myc-driven transcription and cancer cell proliferation. These findings further highlighted the key functional role of Lys31/57 in modulating the DNA binding activity of Max. Intrigued by these results, we envisioned that masking the Lys31 and Lys57 residues with a stimuli-responsive group would yield a caged TF with halted DNA binding activity, which can be reactivated on demand upon a site-selective unmasking of the key Lys residues (Figure 1).

To test our hypothesis, we first aimed to synthesize the DNA binding domain of Max by replacing the Lys31 and Lys57 with Lys residues bearing Nvoc protected ε-amine. The Nvoc group was selected as the caging group owing to its compatibility with SPPS and the relatively mild decaging condition in aqueous buffer.^13^ To this end, we divided the DNA binding domain of Max into two segments to implement the NCL-desulfurization strategy at the Ala58 junction.^14^ We were able to prepare the following segments: Cys-Max(59–93) segment **1** and MaxK31NvocK57Nvoc(13–57)-NHNH_2_ segment **2** via standard Fmoc-SPPS with selective incorporation of Nvoc-protected Lys amino acids at Lys31 and Lys57 (see the SI, Section 4). The N-terminal Ala58 residue at the ligation site was temporally mutated to Cys to enable NCL at this site, and the peptide thioester was generated using the peptide-hydrazide method (see the SI, Section 4).^15^ We isolated both segments **1** and **2** in 23% and 19% yield, respectively, after RP-HPLC purification (see the SI, Section 4). With both segments in hand, segment **2** MaxK31NvocK57Nvoc(13–57)-NHNH_2_ was first converted to acyl-azide using NaNO_2_ in a 6 M Gun·HCl, 0.2 M Na_2_HPO_4_ buffer, pH 3.0, at −15 °C, followed by in situ thioesterification upon the addition of 4-mercaptophenylacetic acid (MPAA) (Figure 2B).^16^ The intermediate thioester was then ligated with segment **1** Cys-Max(59–93) at room temperature for 1 h, after which tris­(2-carboxyethyl)­phosphine hydrochloride (TCEP·HCl) was added. LCMS analysis confirmed the completion of ligation in 1.5 h and RP-HPLC purification afforded the desired ligated product **Max-Nvoc-C** in 43% yield (see the SI, Section 5). The isolated ligated product **Max-Nvoc-C** was then subjected to a desulfurization reaction to convert the mutated Cys back to native Ala using radical initiator 2,2′-azobis­[2-(2-imidazolin-2-yl)­propane]­dihydrochloride (VA044), TCEP, and reduced l-glutathione (GSH).^17^ Unfortunately, the Nvoc protecting group was incompatible with the radical desulfurization conditions, resulting in the significant decomposition of the Nvoc group (see the SI, Section 5). After multiple unsuccessful attempts to optimize the desulfurization step under different conditions (e.g., temperature, additives, and reaction time), we decided to explore an alternative desulfurization-free synthetic strategy to obtain caged Max analogs.^18^

**Figure 2:**
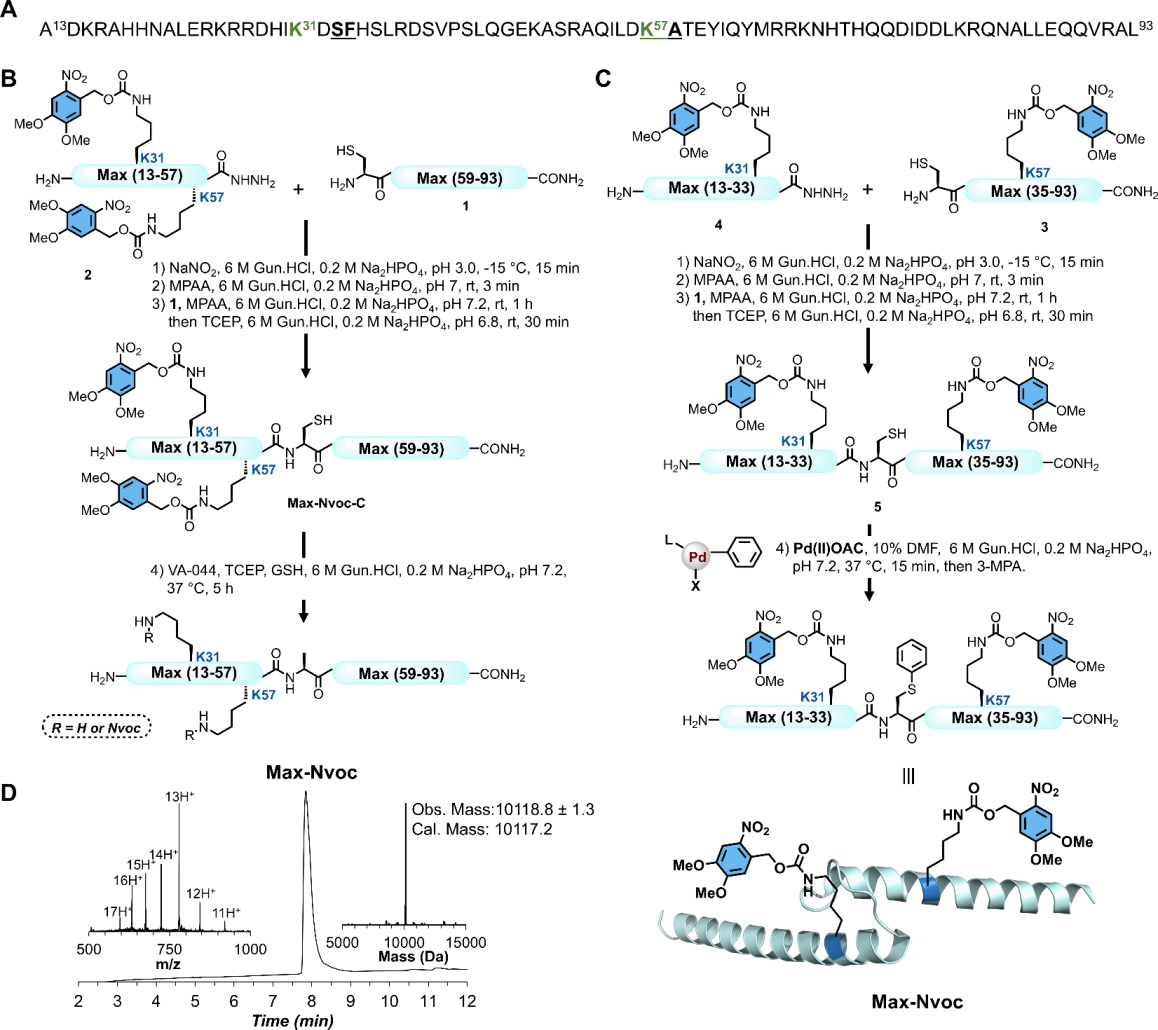
Total chemical synthesis of caged Max transcription factor employing native chemical ligation coupled with palladium-catalyzed C–S cross coupling. (A) Max DNA binding domain sequence (p21 isoform); the acetylation sites are highlighted in green, and the ligation sites (Ser33-Phe34 and Lys57-Ala58) are underlined. (B) Chemical synthesis of **Max-Nvoc** via an NCL-desulfurization approach. (C) Chemical synthesis of **Max-Nvoc** via an NCL and S-arylation strategy. In the Pd­(II)­OAC, the ligand (L) = sSPhos and X = I. (D) LC–MS analysis of isolated **Max-Nvoc** with the observed mass 10118.8 ± 1.3 Da, the calculated mass 10117.2 Da (average isotopes). The ultraviolet (UV) absorbance was monitored at 214 nm, and the *m*/*z* data were acquired over the entire peak.

We have recently reported the power of combining protein synthesis with Pd-mediated S-arylation to enable peptide ligation and diversification at aromatic junctions.^19^ To probe the feasibility of this approach to generate caged Max, we swapped the ligation junction to perform the ligation at Phe34.^19a^ To this end, we prepared the following segments: Cys-MaxK57Nvoc(35–93) segment **3** and MaxK31Nvoc(13–33)-NHNH_2_ segment **4** through standard Fmoc-SPPS. The N-terminal Phe34 residue was mutated to Cys to enable NCL at this site. We obtained both segments **3** and **4** in 15% and 26% yields, respectively, after RP-HPLC purification (see the SI, Section 4). Utilizing these segments, we performed one-pot NCL and S-arylation. Initially, segment **4** MaxK31Nvoc(13–33)-NHNH_2_ was converted to a thioester using NaNO_2_ and MPAA and reacted with segment **3** Cys-MaxK57Nvoc(35–93) in situ to afford the desired ligation product in 1.5 h at room temperature. Subsequently, the reaction mixture was desalted and treated with Pd­(II) oxidative addition complex bearing phenyl residue (Pd­(II)­OAC),^20^ to convert the Cys at the ligation junction into a Phe mimic, yielding the S-arylated product within 15 min (Figure 2C). We were able to isolate the desired final product **Max-Nvoc**, in 29% yield for the three steps after RP-HPLC purification (see the SI, Section 5). The identity and purity of the isolated final product **Max-Nvoc** was confirmed via LC-MS analysis (Figure 2D). The successful isolation of caged Max underscores the effectiveness of combining NCL with late-stage S-arylation chemistry to access photoreactive proteins that are otherwise difficult to obtain using conventional methods.

After successfully isolating **Max-Nvoc**, we investigated the impact of caged Lys31 and Lys57 residues on Max oligomerization and DNA binding activity (Figure 3). First, we incubated **Max-Nvoc** with a dsDNA probe containing the canonical E-box sequence, in the presence or absence of its partner, the Myc oncoprotein that was fluorescently labeled with a 5-carboxytetramethylrhodamine (TAMRA) tag (**T-Myc**, see the SI, section 6). Subsequently, we analyzed the DNA binding activity of the caged Myc/Max and Max/Max oligomers using the electrophoretic mobility-shift assay (EMSA) (see the SI, Section 8).^21^ Remarkably, the DNA binding activity of the caged TFs was significantly reduced, as evidenced by the predominance of free DNA and weak binding, even at higher protein concentrations, for both homo- and heterodimeric complexes, as confirmed by the fluorescent signal of **T-Myc** (Figure 3B–C). Notably, the inhibition of DNA binding was more pronounced for the **Max-Nvoc** homodimer compared with the **T-Myc/Max-Nvoc** heterodimer (Figure 3B). We reasoned that this difference arises from the higher number of masking groups present in the Max homodimer. Furthermore, we envisioned that the substantial inhibition of DNA binding results not only from the neutralization of the critical Lys31/57 residues, but also from the steric hindrance imposed by the bulky Nvoc groups, which can also interfere with TF–DNA interactions. Taken together, these results indicate that the designed caged TF effectively inhibits Max DNA binding activity, providing unprecedented opportunities to control its function through on-demand decaging mechanisms.

**Figure 3:**
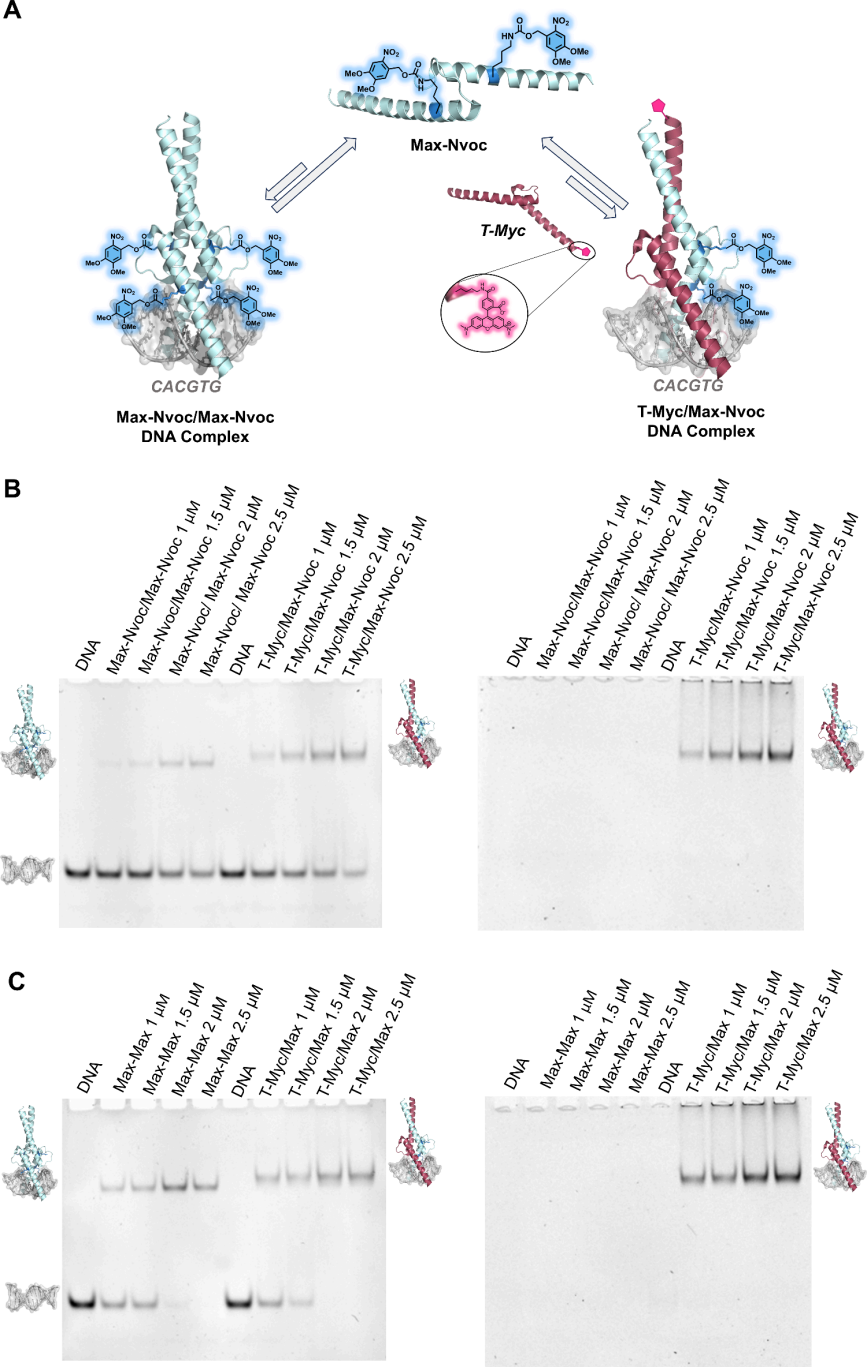
Engineered caged Max suppresses the DNA binding activity of the Max/Max and Myc/Max transcription factor oligomers. (A) Schematic representation of the E-box DNA binding activity of the **Max-Nvoc** homodimer and heterodimer with **T-Myc** (PDB: 1HLO). (B) EMSA experiment of the Max-Nvoc homodimer and heterodimer with **T-Myc** (left; DNA imaging using ethidium bromide, right; **T-Myc** imaging using the fluorescent TAMRA group). (C) Control EMSA experiment of native **Max** homodimer and heterodimer with **T-Myc** (left; DNA imaging using ethidium bromide, right; **T-Myc** imaging using the fluorescent TAMRA group). EMSA conditions: 1 μM DNA probe and 0, 1, 1.5, 2, and 2.5 μM of the homodimer of the Max analog and a heterodimer of Max analog with **T-Myc** in 10 mM MES, 150 mM KCl, 1 mM MgCl_2_, and 10% glycerol buffer (pH 6.0). The EMSA experiments were performed in duplicate.

To examine the decaging and activation of masked Max, we set out to optimize Nvoc removal under aqueous conditions (Figure 4). We initially examined the photoresponsiveness of **Max-Nvoc** under UV irradiation in 10 mM MES, 150 mM KCl, 1 mM MgCl_2_, and 10% glycerol buffer (MES buffer) containing 2 mM 1,4-dithiothreitol (DTT) and 40 mM methoxylamine hydrochloride (CH_3_ONH_2_·HCl) additives.^22^ Subsequently, the reaction mixture was subjected to UV irradiation of 365 nm at room temperature (Figure 4A-B). These conditions led to ∼95% conversion to the desired product decaged **Max-Nvoc** product after 1 h, as confirmed by LC-MS analysis. Further optimization of the decaging conditions, for example irradiation at 350 nm, resulted in 99% conversion to the unmasked product (Figure 4C). Remarkably, similar outcomes were obtained upon the systematic removal of reaction additives and when performed in 1× PBS buffer (see the SI, Section 7).

**Figure 4:**
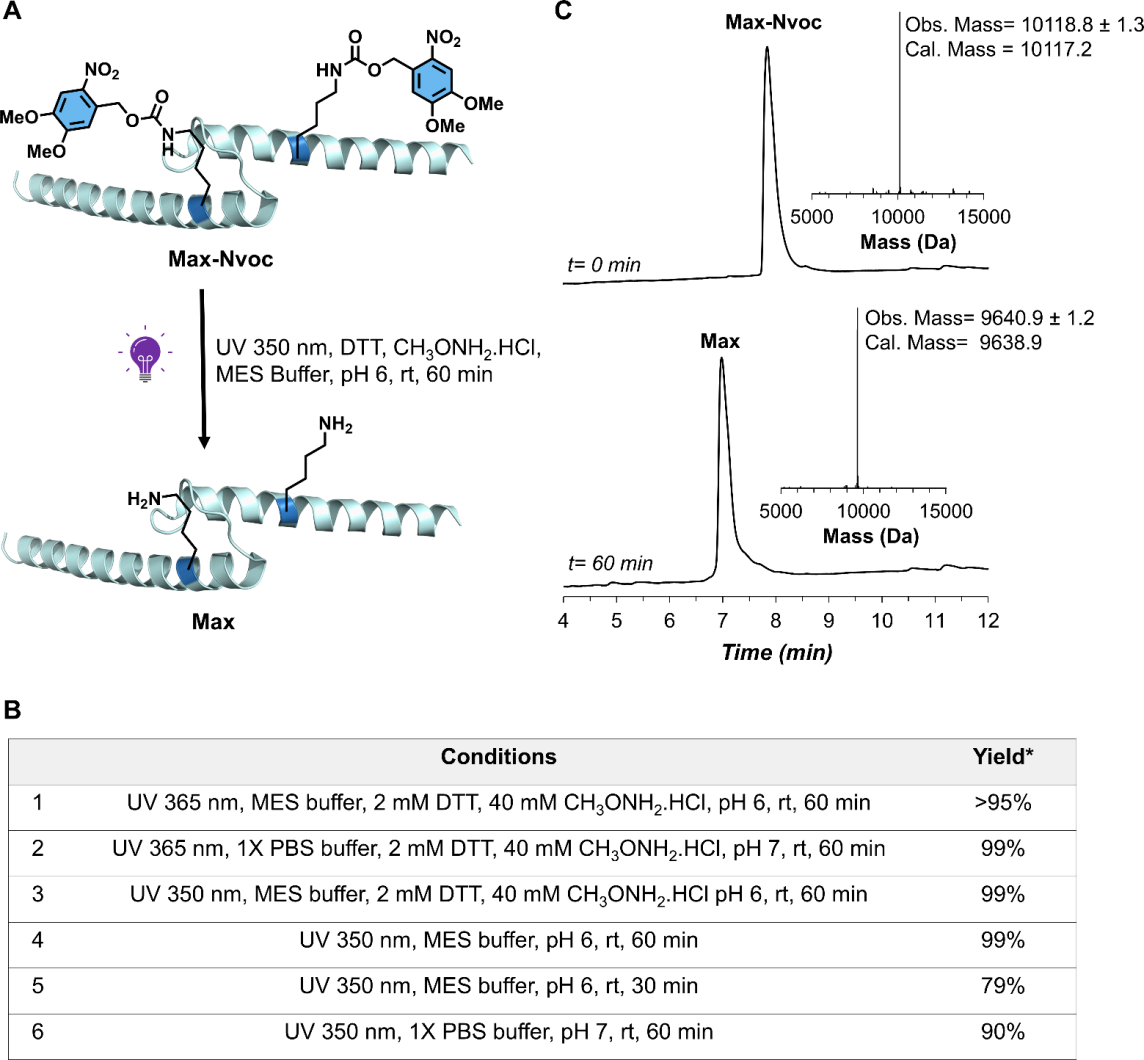
Light-mediated unmasking of caged **Max-Nvoc**. (A) Schematic representation of **Max-Nvoc** unmasking by UV irradiation (PDB: 1HLO). (B) Table summarizing the Nvoc decaging conditions and conversion yields. *Conversion yields were determined by LC-MS analysis. (C) Crude LC-MS analysis of the Nvoc decaging reaction; Conditions: UV 350 nm, MES buffer, 2 mM DTT, 40 mM CH_3_ONH_2_.HCl pH 6, rt, 60 min.

After optimizing the decaging conditions, we next probed the DNA-binding activity of decaged **Max-Nvoc** toward the E-box DNA. We initially incubated the decaged **Max-Nvoc** with the E-box DNA probe and analyzed DNA binding by EMSA (see SI, Section 8). However, under these conditions, we observed a weak association between decaged **Max-Nvoc** and the DNA (see SI, Figure S16). We reasoned that the reduction in the DNA binding activity of Max arises due to the interference of decomposed Nvoc byproducts, e.g., 4,5-dimethoxy-2-nitrosobenzaldehyde, and/or the generation of reactive oxygen species generated during UV decaging, which can interfere with DNA integrity.^23^ Importantly, LC-MS analysis verified the formation of the decaged Max product, supporting our hypothesis that potential oligonucleotide alterations influence DNA binding using the optimized additive-free irradiation. Remarkably, the use of quenchers such as DTT and CH_3_ONH_2_ restored the DNA binding of decaged Max. Under optimized UV irradiation and binding conditions, we validated that on-demand decaging of engineered Max fully reactivates its functional activity, as demonstrated by a clear dose-dependent shift in EMSA (Figure 5B). Intrigued by these results, we probed the impact of the Nvoc group on the folding pattern of the Max analogs via circular dichroism (CD) spectroscopy (see the SI, Section 9). The CD spectra of caged and unmasked Max analogs displayed a similar characteristic α-helical pattern, with deep double minima at 208 and 222 nm (Figure 5C). These results confirm that both **Max-Nvoc** and decaged Max adopt a correct α-helical fold, underscoring that incorporating Nvoc moiety did not significantly interfere with the secondary structure of **Max-Nvoc**. Importantly, further CD analysis in the presence of E-box DNA revealed a marked increase in the helicity for the decaged Max, consistent with structural stabilization, whereas **Max-Nvoc** showed minimal changes due to its weak DNA binding activity, thus supporting the EMSA results (Figure 5C). This inhibition of DNA binding is attributed to the masking and neutralization of the essential Lys31/57 residues, as well as the bulky nature of the Nvoc groups, which can interfere with Max–DNA interactions (Figure 5D). These findings further confirm that **Max-Nvoc** functionality and DNA binding activity were restored following the decaging step. Importantly, these results indicate that the Nvoc group interferes with DNA binding through protein–DNA contact and not due to structural alteration.

**Figure 5:**
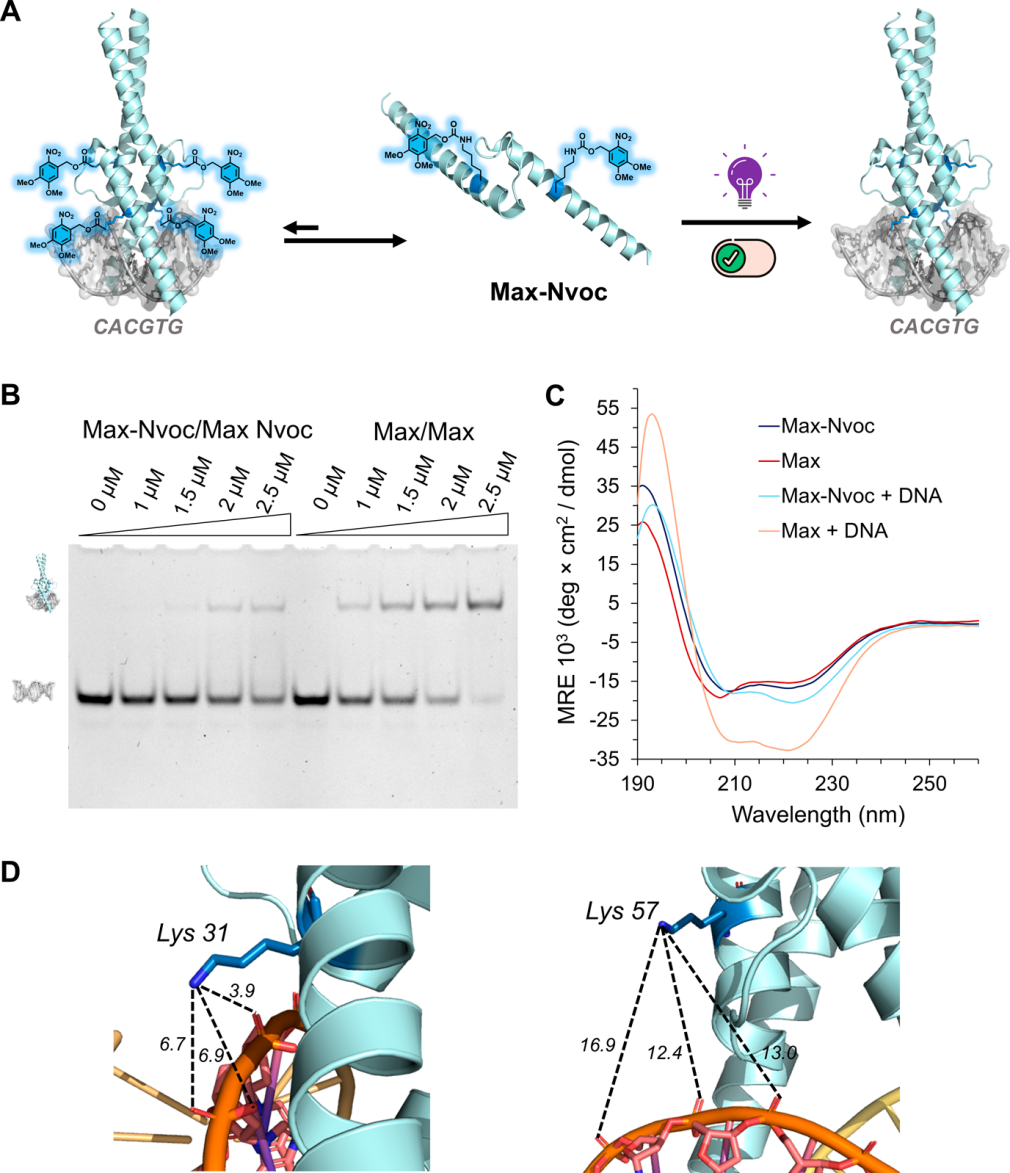
Light-mediated on-demand activation and DNA binding of synthetic **Max.** (A) Schematic representation of the light-triggered DNA binding activity of **Max-Nvoc**. (B) EMSA experiment of **Max-Nvoc** and decaged Max with E-box DNA; Decaging conditions: UV 350 nm, MES buffer, 2 mM DTT, 40 mM CH_3_ONH_2_.HCl pH 6, rt, 60 min. (C) CD analysis of synthetic **Max-Nvoc** and decaged **Max** in the presence and absence of E-box DNA; Decaging conditions: UV 350 nm, MES buffer, 2 mM DTT, 40 mM CH_3_ONH_2_.HCl pH 6, rt, 60 min. (D) Schematic illustration showing interactions between Lys31/57 residues and the phosphate diester groups of the DNA backbone (PDB: 1HLO). EMSA conditions: 1 μM DNA probe and 0, 1, 1.5, 2, and 2.5 μM homodimer of Max analog incubated in 10 mM MES, 150 mM KCl, 1 mM MgCl_2_, and 10% glycerol buffer (pH 6.0). CD conditions: 5 μM homodimer of Max analog in 10 mM MES, 150 mM KCl, 1 mM MgCl_2_, and 10% glycerol buffer (pH 6.0). All CD analyses were performed in triplicate, and the EMSA experiments were performed in duplicate.

We also quantified the DNA binding activity of caged and unmasked Max analogs via biolayer interferometry (BLI) and determined the dissociation constant (K_D_) to the E-box DNA probe (see the SI, Section 10). **Max-Nvoc** exhibited a K_D_ value of 161 μM, highlighting the significant reduction in the DNA binding activity. Remarkably, upon UV irradiation and decaging, the resulting decaged Max exhibited a K_D_ value of 13 nM, which is in line with earlier reports for the native Max and E-box DNA (Figure 6A).^11a,19a,24^ Taken together, these experiments further support the effective functional recovery of caged Max upon unmasking. Finally, we examined the in situ activation of Max-Nvoc in the presence of DNA. We incubated **Max-Nvoc** with E-box DNA probe and then subjected it to 350 nm UV irradiation for varying time intervals ranging from 0 to 70 min, followed by DNA binding analysis via EMSA (Figure 6B). We observed a progressive increase in DNA binding activity with longer UV irradiation times, confirming the effective decaging of the Nvoc group and the consequent functional reactivation of **Max-Nvoc** in the presence of DNA. Taken together, these results corroborate the successful design and synthesis of the light-responsive caged TF, which displays halted DNA binding activity in its caged state and restores DNA binding upon decaging, resembling the activity of its native form upon on-demand activation.

**Figure 6:**
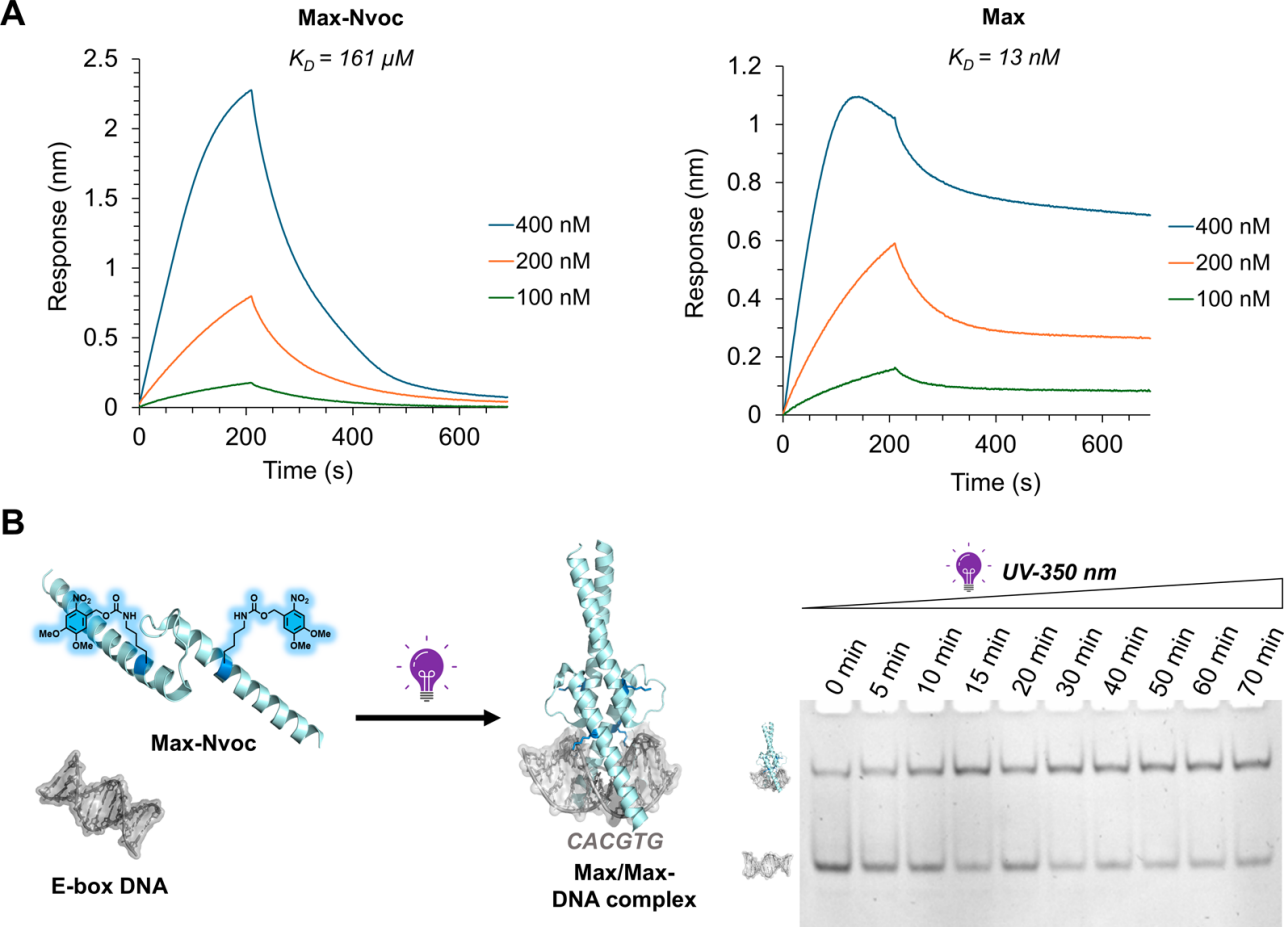
In-situ activation and DNA binding of engineered **Max**. (A) Sensorgrams from biolayer interferometry (BLI) analysis of the binding of **Max-Nvoc** and decaged **Max** to the E-box DNA probe and the calculated K_D_ values for **Max-Nvoc,** K_D_ = 161 μM (R^2^ = 0.99) and **Max,** K_D_ = 13 nM (R^2^ = 0.98); Decaging conditions: UV 350 nm, MES buffer, 2 mM DTT, 40 mM CH_3_ONH_2_.HCl pH 6, rt, 60 min. (B) EMSA analysis of **Max-Nvoc** decaging in the presence of DNA. EMSA conditions: 1 μM DNA probe and 5 μM protein subjected to UV irradiation for varying time intervals ranging from 0 to 70 min; Decaging conditions: UV 350 nm, MES buffer, 2 mM DTT, 40 mM CH_3_ONH_2_·HCl pH 6, rt. The EMSA experiments were performed in duplicate.

## Conclusion

Engineering proteins switches that activate functional outputs on demand provide a versatile strategy to manipulate cellular processes for both fundamental research and therapeutic applications. In this work, we have engineered a light-responsive TF, **Max-Nvoc**, by masking key residues (Lys31 and Lys57) essential for DNA binding. Masking the key residues with the light-responsive Nvoc group enabled the controlled decaging and activation of Max on demand. Remarkably, although the preparation of photoreactive TFs was incompatible with the traditional NCL–desulfurization approach, the developed strategy, employing NCL and palladium-catalyzed C–S cross-coupling, further highlights the power of combining total protein synthesis with late-stage transformations to generate novel, complex modified proteins.^25^ Our reported approach overcame the incompatibility of the photoreactive group with free-radical desulfurization and underscores the importance of developing orthogonal transformations to access uniquely modified proteins.^26^ In addition to combining NCL with S-arylation, we anticipate that other desulfurization-free ligation strategies—such as Ser/Thr ligation, α-ketoacid–hydroxylamine ligation, and diselenide–selenoester ligation—will also be valuable for generating synthetic proteins bearing photoreactive groups.^27^ Importantly, comprehensive DNA binding analyses confirmed that the caged TF exhibits minimal DNA binding activity, which can be efficiently restored on demand. Importantly, the decaging process restores native-like DNA binding activity within minutes upon UV irradiation. Our results substantiate the successful design, synthesis, and activation of the caged Max TF, further highlighting the feasibility of generating novel modified proteins with tailored functions to probe and control essential TF-DNA interactions.^28^ Translating this level of control to complex biological settings will require the development of biocompatible photolabile protecting groups.^29^ This and other research programs are currently being explored in our laboratory.

## Supplementary Material



## Notes

Synthesis and methods, biochemical and biophysical characterization of the synthesized compounds (PDF)
