# Supplementary material for: Engineering Light-Responsive Transcription Factors via Strategic Masking of Post-translational Modification Residues
Source: Bioconjug Chem. 2026 Jan 27;37(2):332–40. doi: 10.1021/acs.bioconjchem.5c00561 (PMC12921659; doi:10.1021/acs.bioconjchem.5c00561)
Supplement: Supplementary file 1 [file bc5c00561_si_001.pdf]

Supporting Information

Engineering Light-Responsive Transcription Factors via Strategic Masking of Post-translational Modification Residues

Raj V. Nithun,[1+] Shada Khoury,[1+] and Muhammad Jbara[1]*

[1]School of Chemistry, Raymond and Beverly Sackler Faculty of Exact Sciences, Tel Aviv University, Tel Aviv, 69978 Israel.

[+]Authors contributed equally

*Correspondence to: jbaram@tauex.tau.ac.il

Table of Contents

1. EXPERIMENTAL **3**

1.1 Materials 3
1.2 LC-MS analysis 4
1.3 Preparative RP-HPLC purification 4
1.4 pH measurements for ligation reactions 4
1.5 UV irradiation 4

2. SYNTHESIS OF PALLADIUM (II) OXIDATIVE ADDITION COMPLEX (PD(II)OAC) **5**

3. PROTEIN AND DNA SEQUENCES **5**

4. CHEMICAL SYNTHESIS OF THE PEPTIDE SEGMENTS **6**

4.1 Preparation of 2-chlorotrityl-hydrazino-resin 6
4.2 Synthesis of segment 1 Cys-Max(59-93) 6
4.3 Synthesis of segment 2 MaxK31NvocK57Nvoc(13-57)-NHNH$_2$ 8
4.4 Synthesis of segment 3 Cys-MaxK57Nvoc(35-93) 9
4.5 Synthesis of segment 4 MaxK31Nvoc(13-33)-NHNH$_2$ 11
4.6 Synthesis of segment M1 Cys-Myc(400-434)-T 12
4.7 Synthesis of segment M2 Myc(353-398)-NHNH$_2$ 14

5. CHEMICAL SYNTHESIS OF CAGED MAX **15**

5.1 Chemical synthesis of Max-Nvoc via native chemical ligation (NCL)-desulfurization 15
5.2 Chemical synthesis of Max-Nvoc via NCL and S-arylation 19

6. CHEMICAL SYNTHESIS OF T-MYC AND NATIVE MAX **21**

6.1 Chemical synthesis of T-Myc via NCL-desulfurization 21
6.2 Chemical synthesis of native Max. 22

7. DECAGING OF MAX-NVOC ANALOG **23**

8. DNA-BINDING ANALYSIS AND ELECTROPHORETIC MOBILITY-SHIFT ASSAY (EMSA) **25**

9. CIRCULAR DICHROISM (CD) ANALYSIS **28**

10. OCTET BIOLAYER INTERFEROMETRY BINDING ASSAY (BLI) **29**

11. REFERENCES **30**

1. Experimental

1.1 Materials

Fmoc-L-Phe-OH, Fmoc-L-Asn(Trt)-OH, Fmoc-L-Gln(Trt)-OH, Fmoc-L-Arg(Pbf)-OH, Fmoc-L-Tyr(tBu)-OH, Fmoc-L-Glu(OtBu)-OH, Fmoc-L-Val-OH, Fmoc-L-Ala-OH, Fmoc-L-Leu-OH, Fmoc-L-His(Trt)-OH, Fmoc-L-Asp(OtBu)-OH, Fmoc-L-Pro-OH, Fmoc-L-Cys(Trt)-OH, Fmoc-L-Lys(Boc)-OH, Fmoc-L-Ile-OH, Fmoc-L-Thr(tBu)-OH, Fmoc-L-Ser(tBu)-OH, Fmoc-Gly-OH, Fmoc-L-Nle-OH, Tetrakis(triphenylphosphine)palladium, Boc-L-Cys(Trt)-OH, SPhos ligand, Methoxyamine hydrochloride, DL-Dithiothreitol (DTT), Tris(2-carboxyethyl)phosphine hydrochloride (TCEP), 2-Methyl-2-propanethiol, L-Glutathione reduced (GSH), Sodium nitrite and Ethidium bromide were purchased from Sigma-Aldrich. 2,2'-Azobis(2-methylpropionamidine)dihydrochloride (VA044) was purchased from TCI-Chemicals. Fmoc-L-Lys(Nvoc)-OH and 4-Mercaptophenylacetic acid (MPAA) were purchased from S.L-Moran. 1-[Bis(dimethylamino)methylene]-1H-1,2,3-triazolo[4,5-b]pyridinium3-oxid hexafluorophosphate (HATU), (2-(1H-benzotriazol-1-yl)-1,1,3,3-tetramethyluronium hexafluorophosphate (HBTU), and HOBt hydrate were purchased from Luxembourg Bio Technologies Ltd. HO-TCP(Cl)-ProTide Resin and Rink Amide ProTide resin were obtained from CEM. Oligonucleotides were purchased from Integrated DNA Technologies (IDT, Coralville, IA). Trifluoroacetic acid (TFA, ≥99% ReagentPlus®), diisopropylethylamine (DIEA, ≥99% ReagentPlus®), piperidine (≥99% ReagentPlus®), triisopropylsilane (TIS, 98%), formic acid (98-100% for LC/MS), Diethyl ether (Et_2O, 99.8% stabilized, ACS grade), Dichloromethane (CH_2Cl_2, ≥99.5% stabilized with 50 ppm Amylene), Peptide Synthesis-grade N,N-dimethylformamide (DMF), Acetonitrile (LC/MS, HPLC Grade) and dimethyl sulfoxide (DMSO, ≥99.5% ReagentPlus®) were purchased from Bio-Lab ltd. TBE Running buffer (5X), Acrylamide Solution (40%), Tetramethylethylenediamine (TEMED), Ammonium Persulfate (APS), and 6X DNA Loading Dye were purchased from Thermo Fisher Scientific. Water for all reactions carried out on proteins and reverse-phase purification was obtained via deionized water filtration through a MilliporeSigmaTM Milli-QTM Ultrapure Water System. All chemicals obtained from the supplier were used as received without further purification.

1.2 LC-MS analysis

Analytical LC was acquired using Thermo Scientific Vanquish HPLC, Mobile phases used are solvent A (0.05% formic acid in water) and solvent B (0.05% formic acid in acetonitrile) and mass spectrometry using Thermo Scientific ISQ EM Mass spectrometer.

Method A: bioZen™ 2.6 µm-C4 Widepore LC column (150 x 2.1 mm); LC conditions: 5% B from 0–1.0 min, then a linear gradient from 5% to 50% B from 1.0–11.0 min (i.e. 4.5% per min), 0.3 mL/min flow rate.

Method B: XBridge® Protein BEH 2.5 µm-C4 300 Å LC column (150 x 2.1 mm); LC conditions: 5% B from 0–1.0 min, then a linear gradient from 5% to 50% B from 1.0–11.0 min (i.e. 4.5% per min), 0.3 mL/min flow rate.

1.3 Preparative RP-HPLC purification

Preparative RP-HPLC was performed using Thermo Scientific DIONEX UltiMate 3000 Variable Wavelength Detector, equipped with an XBridge® Protein BEH C4 OBD™ Prep column, 300 Å, 5 µm, (250 x 10 mm). Mobile phases used for LC analysis were solvent A (0.05% TFA in water), and solvent B (0.05% TFA in acetonitrile). The following LC methods were used:

Method A: 5% B from 0–5 min, then a linear gradient from 5% to 60% B from 5-60 min (i.e. 1% per min), 4 mL/min flow rate at 30 °C.

Method B: 5% B from 0–5 min, then a linear gradient from 5% to 20% B from 5-10 min, followed by another linear gradient from 20% to 60% B from 10-50 min (i.e. 1% per min), 4 mL/min flow rate at 30 °C.

1.4 pH measurements for ligation reactions

All pH values in aqueous 6 M Gun·HCl were determined using a VWR pH meter with a SENTEK electrode.

1.5 UV irradiation

The UV irradiation reactions were carried out using a Rayonet photochemical chamber mini reactor (RMR-600) equipped with eight Rayonet lamps (350 nm, 4 W), each 6 inches in length.

Note: No unexpected or unusually high safety hazards were encountered during the experiments.

2. Synthesis of palladium (II) oxidative addition complex (Pd(II)OAC)

This compound was prepared according to literature procedure.[1–3] The ^{1}H and ^{13}C NMR spectra of the obtained material are identical to those reported in literature.[1]

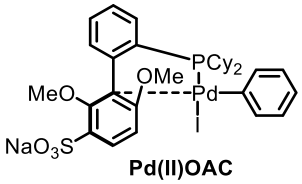

Pd(II)OAC

3. Protein and DNA sequences

Max(13-93):

ADKRAHHNALERKRRDHI**K^{31}**D**SF**HSLRDSVPSLQGEKASRAQILD**K^{57}A**TEYIQYMRRKNHTHQQDIDDLKRQNALLEQQVRAL

The Phe34 and Ala58 were mutated to Cys to enable native chemical ligation at these positions in their respective synthetic strategies. The Met65 was replaced with the isologous norleucine (Nle) residue to avoid Met oxidation.

Myc(353-434):

NVKRRTHNVLERQRRNELKRSFFALRDQIPELENNEKAPKVVILK**KA**TAYILSVQAEEQKLISEEDLLRKRREQLKHKLEQL

E-box DNA probe:

5’-CCGGCTGA*CACGTG*GTATTAAT-3’

4. Chemical synthesis of the peptide segments

4.1 Preparation of 2-chlorotrityl-hydrazino-resin

The preparation was carried out by the following scheme.

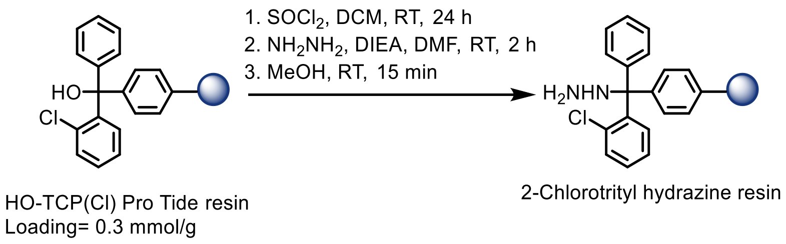

333.3 mg HO-TCP(Cl) resin (loading = 0.3 mmol/g, 0.1 mmol) were mixed with $SOCl_2$ (336 µL, 4.6 mmol, 1.5 M) and 3 ml of DCM in a septum-capped oven-dried round-bottom-flask that was cooled with N_2. The mixture was stirred overnight. $SOCl_2$ (134 µL, 1.8 mmol) was added the following morning and mixed for another 6 hours. The resin was then transferred into a fritted syringe and washed with DCM (8 mL X 6), DMF (8 mL X 3), and cooled to 0 °C. A mixture of DIEA (190 µl, 1.1 mmol, 1.3 M) and hydrazine hydrate-50% (96 µl, 3.1 mmol, 3.6 M) in DMF (571 µl) was added slowly. Then, the suspension was stirred at room temperature. After 2 h, 115 µl MeOH was added to the reaction mixture to ensure the unreacted sites on the resin were capped and stirred for an additional 15 min. Finally, the resin was washed with DMF, H_2O, DMF, MeOH, and Et_2O and dried under a vacuum.[4]

4.2 Synthesis of segment 1 Cys-Max(59-93)

The synthesis was carried out according to the following scheme:

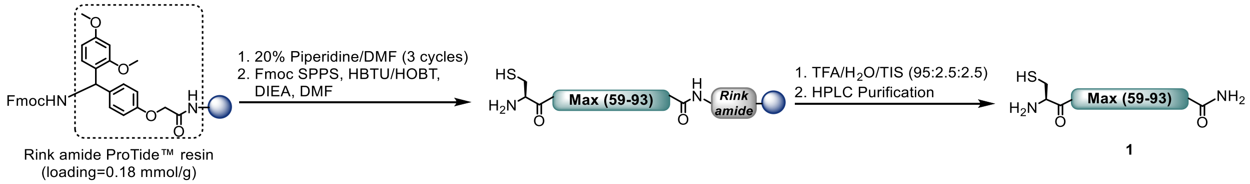

The synthesis of peptide segment **1** was carried out using stepwise Fmoc-SPPS on Rink amide ProTide™ resin (278 mg, loading 0.18 mmol/g, 0.05 mmol scale). The resin was pre-swollen in DMF for 30 min and then treated with 20% piperidine and 0.05% formic acid in DMF to remove the Fmoc protecting group and then coupled residues 93-78 in a stepwise fashion with Fmoc-amino acid (10 equiv., 0.5 mmol, 0.21 M), using HBTU/HOBt (10 equiv., 0.5 mmol, 0.21 M) and DIEA (20 equiv., 1 mmol, 0.42 M) in 2 mL DMF. The coupling was carried out manually at 30 °C for 20 minutes coupling time. Subsequently, the resin was transferred to the CSBio automated peptide synthesizer and residues 77-58 were added in a stepwise

fashion with Fmoc-amino acid (10 equiv., 0.5 mmol, 33.3 mM), using HBTU/HOBt (10 equiv., 0.5 mmol, 33.3 mM) and DIEA (20 equiv., 1 mmol, 66.7 mM). The coupling was carried out at 30 °C for 45 minutes coupling time. The resin was washed with DMF (5 mL x 3), MeOH (5 mL x 3), and DCM (5 mL x 3) and dried under a vacuum. To remove side chain protecting groups and release the peptide chains, a mixture of TFA/H_2O/TIS (95:2.5:2.5, 7 mL for 0.025 mmol scale) was added to each peptide resin and shaken for 3.5 h at RT. The resin was removed by filtration and washed with TFA (2 × 1 mL). To precipitate the peptide, the combined filtrate was added dropwise to cold diethyl ether (25 mL for 0.025 mmol resin) followed by centrifugation at 4000 rpm for 7 min. Then, the diethyl ether was decanted, followed by the dissolution of the peptide in 50% acetonitrile/water, diluted to 25% acetonitrile/water, and lyophilized to get crude segment **1** Cys-Max(59-93) (141.8 mg, 31.9 µmol) as a white powder. The crude dry peptide powder was purified by RP-HPLC (Method B described in Section 1.3) affording the product Cys-Max(59-93) (50.2 mg, 11.3 µmol, 23% yield based on 0.05 mmol resin) as a white powder.

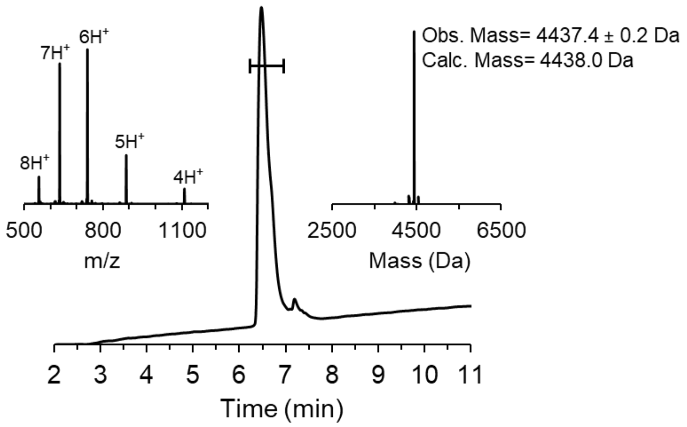

Figure S1. LC-MS analysis of segment **1** Cys-Max(59-93) with the observed mass 4437.4 ± 0.2 Da, calculated mass 4438.0 Da (average isotopes). The UV absorbance was monitored at 214 nm, and the mass-to-charge (m/z) data was acquired over the marked region in the chromatogram. LC-MS analysis was carried out with Method A depicted in section 1.2

4.3 Synthesis of segment 2 MaxK31NvocK57Nvoc(13-57)-$NHNH_2$

The synthesis was carried out according to the following scheme:

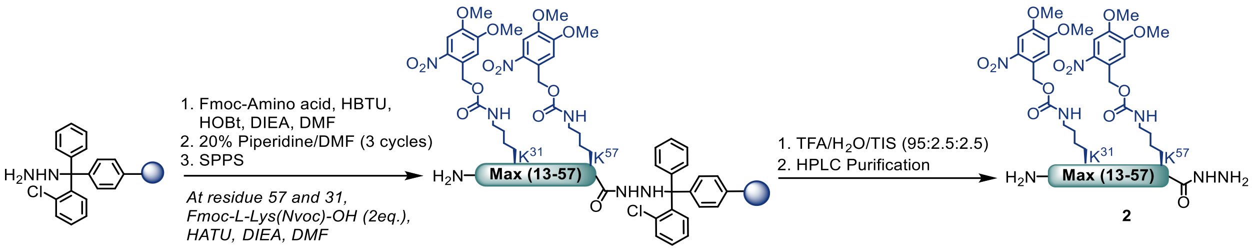

The synthesis of segment **2** MaxK31NvocK57Nvoc(13-57)-$NHNH_2$ was carried out using stepwise Fmoc-SPPS on hydrazide resin (167 mg, loading 0.3 mmol/g, 0.05 mmol scale). The resin was pre-swollen in DMF for 30 min and then, coupled Fmoc-L-Lys(Nvoc)-OH (2 equiv., 0.1 mmol, 50 mM) using HATU (2 equiv., 0.1 mmol, 50 mM) and DIEA (4 equiv., 0.2 mmol, 100 mM) in 2 mL DMF for 1 h. Residues 56-47 was then coupled in a stepwise fashion with Fmoc-amino acid (10 equiv., 0.5 mmol, 0.21 M), using HBTU/HOBt (10 equiv., 0.5 mmol, 0.21 M) and DIEA (20 equiv., 1 mmol, 0.42 M) in 2 mL DMF. The coupling was carried out manually at 30 °C for 20 minutes coupling time. After, the resin was transferred to the CSBio automated peptide synthesizer and residues 46-32 were added in a stepwise fashion with Fmoc-amino acid (10 equiv., 0.5 mmol, 33.3 mM), using HBTU/HOBt (10 equiv., 0.5 mmol, 33.3 mM) and DIEA (20 equiv., 1 mmol, 66.7 mM). The coupling was carried out at 30 °C for 45 minutes coupling time. The synthesis was carried out until 31st residue, then the resin was taken out of the synthesizer and coupled Fmoc-L-Lys(Nvoc)-OH (2 equiv., 0.1 mmol, 50 mM) using HATU (2 equiv., 0.1 mmol, 50 mM) and DIEA (4 equiv., 0.2 mmol, 100 mM) in 2 mL DMF for 1 h. Then, the resin was transferred again back to the CSBio automated peptide synthesizer and residues 30-13 added in a stepwise fashion with Fmoc-amino acid (10 equiv., 0.5 mmol, 33.3 mM), using HBTU/HOBt (10 equiv., 0.5 mmol, 33.3 mM) and DIEA (20 equiv., 1 mmol, 66.7 mM). When the synthesis was completed, the peptide resin was washed with DMF (5 mL x 3), MeOH (5 mL x 3), and DCM (5 ml x 3) and dried under a vacuum. To remove side chain protecting groups and release the peptide chains, a mixture of TFA/H_2O/TIS (95:2.5:2.5, 7 mL for 0.025 mmol scale) was added to the resin which was shaken for 3.5 h at RT. The resin was removed by filtration and washed with TFA (2 × 1 mL). To precipitate the peptide, the combined filtrate was added dropwise to cold diethyl ether (25 mL for 0.025 mmol resin) followed by centrifugation at 4000 rpm for 7 min. Then, the diethyl ether was decanted, followed by the dissolution of the peptide in 50% acetonitrile/water, diluted to 25% acetonitrile/water, and lyophilized to get crude segment **2** MaxK31NvocK57Nvoc(13-57)-$NHNH_2$ (197 mg, 34.5 µmol) as a white powder. The crude dry peptide powder was purified by RP-HPLC (Method B

described in Section 1.3) affording the product MaxK31NvocK57Nvoc(13-57)-$NHNH_2$ (54.2 mg, 9.5µmol, 19% yield based on 0.05 mmol resin) as a white powder.

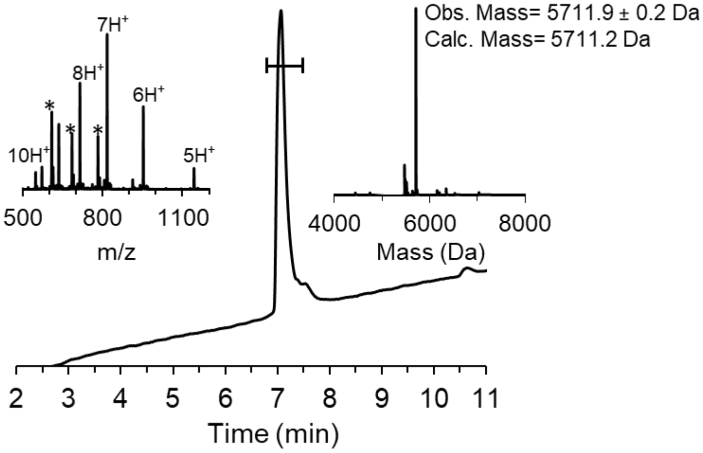

Figure S2. LC-MS analysis of segment **2** MaxK31NvocK57Nvoc(13-57)-$NHNH_2$ with the observed mass 5711.9 ± 0.2 Da, calculated mass 5711.2 Da (average isotopes). * o-nitroveratryloxycarbonyl (Nvoc) group decomposed mass. The UV absorbance was monitored at 214 nm, and the m/z data was acquired over the marked region in the chromatogram. LC-MS analysis was carried out with Method A depicted in section 1.2.

4.4 Synthesis of segment 3 Cys-MaxK57Nvoc(35-93)

The synthesis was carried out according to the following scheme:

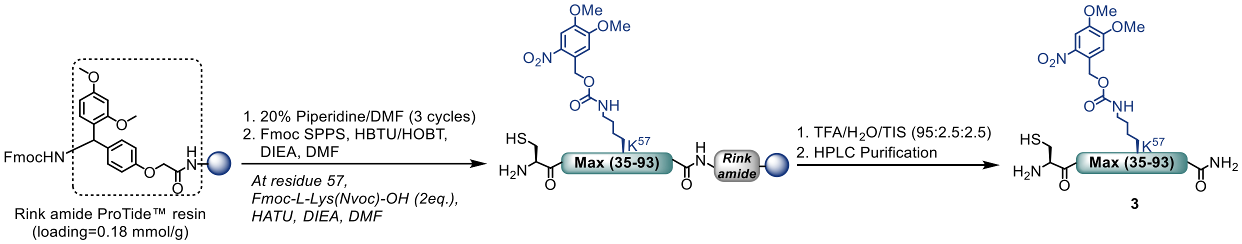

The synthesis of segment **3** Cys-MaxK57Nvoc(35-93) was carried out using stepwise Fmoc-SPPS on Rink amide ProTide™ resin (556 mg, loading 0.18 mmol/g, 0.1 mmol scale). The resin was pre-swollen in DMF for 30 min and then treated with 20% piperidine and 0.05% formic acid in DMF to remove the Fmoc protecting group. Subsequently, the resin was transferred to the CSBio automated peptide synthesizer and residues 93-58 were added in a stepwise fashion with Fmoc-amino acid (10 equiv., 1 mmol, 66.7 mM), using HBTU/HOBt (10 equiv., 1 mmol, 66.7 mM) and DIEA (20 equiv., 2 mmol, 0.13 M). The coupling was carried out at 30 °C for 45 minutes coupling time. The resin was then taken out of the synthesizer and coupled manually with Fmoc-L-Lys(Nvoc)-OH (2 equiv., 0.2 mmol, 100 mM) using HATU (2 equiv., 0.2 mmol, 100 mM) and DIEA (4 equiv., 0.4 mmol, 200 mM) in 2 mL DMF for 1 h. Then, the resin was

transferred back to the CSBio automated peptide synthesizer and residues 56-34 added in a stepwise fashion with Fmoc-amino acid (10 equiv., 1 mmol, 66.7 mM), using HBTU/HOBt (10 equiv., 1 mmol, 66.7 mM) and DIEA (20 equiv., 2 mmol, 0.13 M). When the synthesis was completed, the peptide resin was washed with DMF (5 mL x 3), MeOH (5 mL x 3), and DCM (5 ml x 3) and dried under vacuum. To remove side chain protecting groups and release the peptide chains, a mixture of TFA/H_2O/TIS (95:2.5:2.5, 7 mL for 0.025 mmol scale) was added to the resin which was shaken for 3 h at RT. The resin was removed by filtration and washed with TFA (2 × 1 mL). To precipitate the peptide, the combined filtrate was added dropwise to cold diethyl ether (25 mL for 0.025 mmol resin) followed by centrifugation at 4000 rpm for 7 min. Then, the diethyl ether was decanted, followed by the dissolution of the peptide in 25% acetonitrile/water and lyophilized to get crude Cys-MaxK57Nvoc(35-93) (556 mg, 76.5 µmol) as a white powder. The crude dry peptide powder was purified by RP-HPLC (Method B described in Section 1.3), affording the product Cys-MaxK57Nvoc(35-93) (107.3 mg, 14.8 µmol, 15% yield based on 0.1 mmol resin) as a white powder.

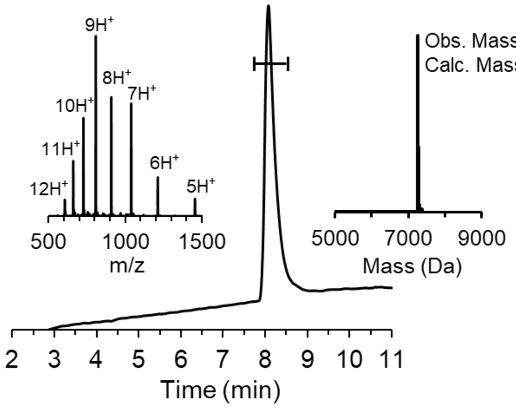

Figure S3. LC-MS analysis of segment **3** Cys-MaxK57Nvoc(35-93) with the observed mass 7267.6 ± 0.2 Da, calculated mass 7266.1 Da (average isotopes). The UV absorbance was monitored at 214 nm, and the m/z data was acquired over the marked region in the chromatogram. LC-MS analysis was carried out with Method B depicted in section 1.2.

4.5 Synthesis of segment 4 MaxK31Nvoc(13-33)-$NHNH_2$

The synthesis was carried out according to the following scheme:

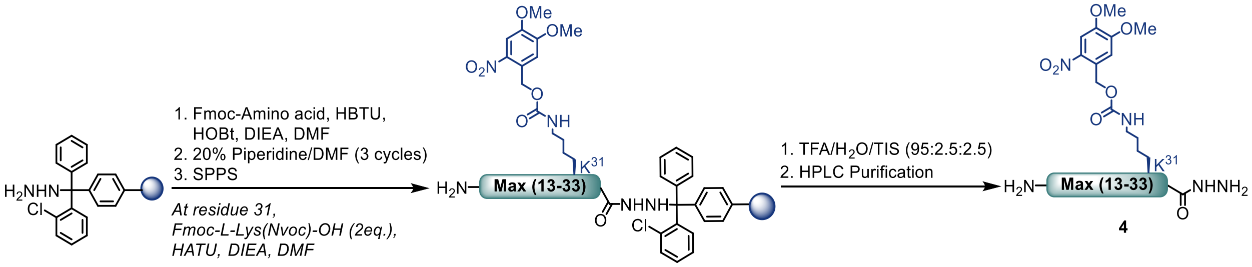

The synthesis of segment **4** MaxK31Nvoc(13-33)-$NHNH_2$ was carried out using stepwise Fmoc-SPPS on hydrazide resin (167 mg, loading 0.3 mmol/g, 0.05 mmol scale). The resin was pre-swollen in DMF for 30 min and then coupled residues 33 and 32 in a stepwise fashion with Fmoc-amino acid (5 equiv., 0.25 mmol, 125 mM), using HBTU/HOBt (5 equiv., 0.25 mmol, 125 mM) and DIEA (10 equiv., 0.5 mmol, 250 mM) in 2 mL DMF. The coupling was carried out manually at 30 °C for 30 minutes coupling time. Then, the resin was then coupled with Fmoc-L-Lys(Nvoc)-OH (2 equiv., 0.1 mmol, 50 mM) using HATU (2 equiv., 0.1 mmol, 50 mM) and DIEA (4 equiv., 0.2 mmol, 100 mM) in 2 mL DMF for 1 h. After, the resin transferred to the CSBio automated peptide synthesizer and residues 30-13 added in a stepwise fashion with Fmoc-amino acid (10 equiv., 0.5 mmol, 33.3 mM), using HBTU/HOBt (10 equiv., 0.5 mmol, 33.3 mM) and DIEA (20 equiv., 1 mmol, 66.7 mM). The coupling was carried out at 30 °C for 45 minutes coupling time. Finally, the peptide resin was washed with DMF (5 mL x 3), MeOH (5 mL x 3), and DCM (5 ml x 3) and dried under vacuum. To remove side chain protecting groups and release the peptide chains, a mixture of TFA/H_2O/TIS (95:2.5:2.5, 7 mL for 0.025 mmol scale) was added to the resin which was shaken for 3 h at RT. The resin was removed by filtration and washed with TFA (2 × 1 mL). To precipitate the peptide, the combined filtrate was added dropwise to cold diethyl ether (25 mL for 0.025 mmol resin) followed by centrifugation at 4000 rpm for 7 min. Then, the diethyl ether was decanted, followed by the dissolution of the peptide in 25% acetonitrile/water and lyophilized to get crude MaxK31Nvoc(13-33)-$NHNH_2$ (124 mg, 44.2 µmol) as a white powder. The crude dry peptide powder was purified by RP-HPLC (Method A described in Section 1.3), affording the product MaxK31Nvoc(13-33)-$NHNH_2$ (36 mg, 12.8 µmol, 26% yield based on 0.05 mmol resin) as a white powder.

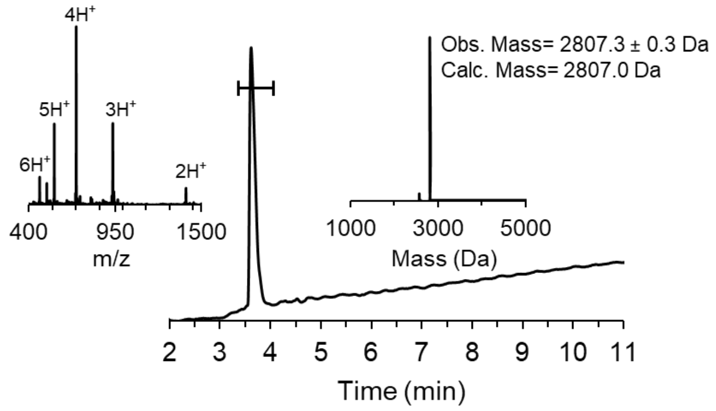

Figure S4. LC-MS analysis of segment **4** MaxK31Nvoc(13-33)-$NHNH_2$ with the observed mass 2807.3 ± 0.3 Da, calculated mass 2807.0 Da (average isotopes). The UV absorbance was monitored at 214 nm, and the m/z data was acquired over the marked region in the chromatogram. LC-MS analysis was carried out with Method B depicted in section 1.2.

4.6 Synthesis of segment M1 Cys-Myc(400-434)-T

The synthesis was carried out according to the following scheme:

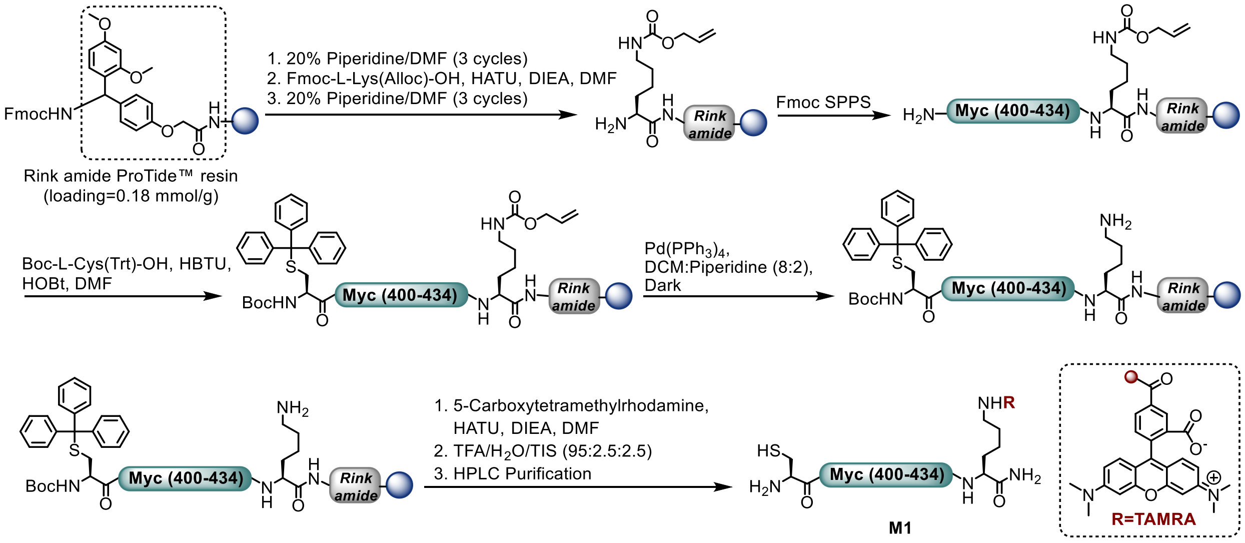

The synthesis of segment **M1** Cys-Myc(400-434)-T was carried out using stepwise Fmoc-SPPS on Rink amide ProTide™ resin (278 mg, loading 0.18 mmol/g, 0.05 mmol scale). The resin was pre-swollen in DMF for 30 min and then treated with 20% piperidine and 0.05% formic acid in DMF to remove the Fmoc protecting group. Subsequently, the functionalized resin was coupled with Fmoc-L-Lys(Alloc)-OH (4 equiv., 0.2 mmol, 0.1 M), using HATU (4 equiv., 0.2 mmol, 0.1 M) and DIEA (8 equiv., 0.4 mmol, 0.2 M) in 2 mL DMF for 2 h. The resin was then transferred to the CSBio automated peptide synthesizer and residues

434-400 were added in a stepwise fashion with Fmoc-amino acid (10 equiv., 0.5 mmol, 33.3 mM), using HBTU/HOBt (10 equiv., 0.5 mmol, 33.3 mM) and DIEA (20 equiv., 1 mmol, 66.7 mM) per amino acid. The coupling was carried out at room temperature for 45 minutes. Then the resin was taken out of the synthesizer and manually coupled with the last amino acid Boc-L-Cys(Trt)-OH (5 equiv., 0.25 mmol, 0.12 M) using HBTU/HOBt (5 equiv., 0.25 mmol, 0.12 M) and DIEA (10 equiv., 0.5 mmol, 0.24 M). Finally, to remove the Alloc protecting group, the resin was washed with DCM (5 mL x 3) and treated with $Pd(PPh_3)_4$ (1 equiv., 0.05 mmol) in DCM/piperidine (8:2, 2 ml) and shaken for 30 min at 25 °C under exclusion of light. Then, the resin was coupled with 5-Carboxytetramethylrhodamine (TAMRA, 2 equiv., 0.1 mmol, 50 mM) with HATU (2 equiv., 0.1 mmol, 50 mM) and DIEA (4 equiv., 0.2 mmol, 100 mM) for 2 h at RT. After, the resin was washed with DMF (5 mL x 3), MeOH (5 mL x 3), and DCM (5 mL x 3) and dried under a vacuum. To remove side chain protecting groups and release the peptide chains, a mixture of TFA/H_2O/TIS (95:2.5:2.5, 7 mL for 0.025 mmol scale) was added to each peptide resin and shaken for 3.5 h at RT. The resin was removed by filtration and washed with TFA (2 × 1 mL). To precipitate the peptide, the combined filtrate was added dropwise to cold diethyl ether (25 mL for 0.025 mmol resin) followed by centrifugation at 4000 rpm for 7 min. Then, the diethyl ether was decanted, followed by the dissolution of the peptide in 50% acetonitrile/water, diluted to 25% acetonitrile/water, and lyophilized to get crude segment **M1** Cys-Myc(400-434)-T (162 mg, 33.2 µmol) as a pink powder. The crude dry peptide powder was purified by RP-HPLC (Method A described in Section 1.3) affording the product segment **M1** Cys-Myc(400-434)-T (25.3 mg, 5.2 µmol, 10% yield, based on 0.05 mmol resin) as a pink powder.

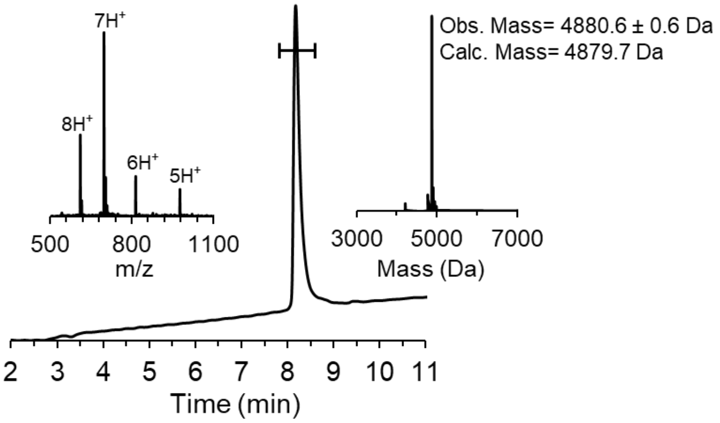

Figure S5. LC-MS analysis of segment **M1** Cys-Myc(400-434)-T with the observed mass 4880.6 ± 0.6 Da, calculated mass 4879.7 Da (average isotopes). The UV absorbance was monitored at 214 nm, and the m/z data was acquired over the marked region in the chromatogram. LC-MS analysis was carried out with Method A depicted in section 1.2.

4.7 Synthesis of segment M2 Myc(353-398)-$NHNH_2$

The synthesis was carried out according to the following scheme:

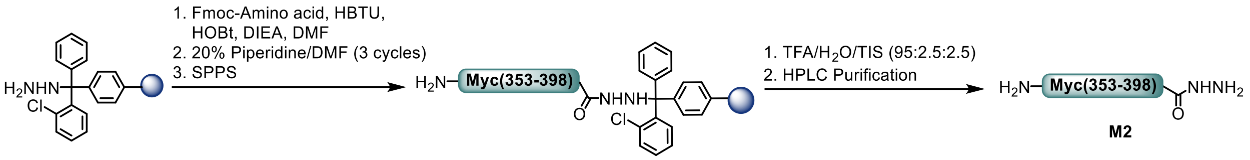

The synthesis of segment **M2** Myc(353-398)-$NHNH_2$ was carried out using stepwise Fmoc-SPPS on hydrazide resin (167 mg, loading 0.3 mmol/g, 0.05 mmol scale). The resin was pre-swollen in DMF for 30 min and then the resin was transferred to the CSBio automated peptide synthesizer and residues 353-398 were added in a stepwise fashion with Fmoc-amino acid (10 equiv., 0.5 mmol, 33.3 mM), using HBTU/HOBt (10 equiv., 0.5 mmol, 33.3 mM) and DIEA (20 equiv., 1 mmol, 66.7 mM). The coupling was carried out at room temperature for 45 minutes coupling time. The resin was washed with DMF (5 mL x 3), MeOH (5 mL x 3), and DCM (5 mL x 3) and dried under a vacuum. To remove side chain protecting groups and release the peptide chains, a mixture of TFA/H_2O/TIS (95:2.5:2.5, 7 mL for 0.025 mmol scale) was added to each peptide resin and shaken for 3.5 h at RT. The resin was removed by filtration and washed with TFA (2 × 1 mL). To precipitate the peptide, the combined filtrate was added dropwise to cold diethyl ether (25 mL for 0.025 mmol resin) followed by centrifugation at 4000 rpm for 7 min. Then, the diethyl ether was decanted, followed by the dissolution of the peptide in 50% acetonitrile/water, diluted to 25% acetonitrile/water, and lyophilized to get crude segment **M2** Myc(353-398)-$NHNH_2$ (270 mg, 48 µmol) as a white powder. The crude dry peptide powder was purified by RP-HPLC (Method A described in Section 1.3) affording the product segment **M2** Myc(353-398)-$NHNH_2$ (59 mg, 10.5 µmol, 21 % yield based on 0.05 mmol resin) as a white powder.

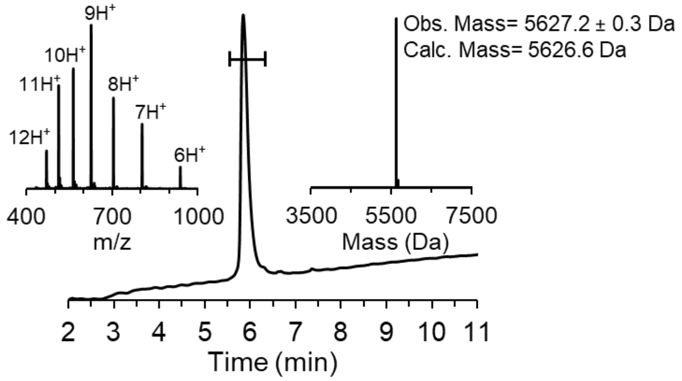

Figure S6. LC-MS analysis of segment **M2** Myc(353-398)-$NHNH_2$ with the observed mass 5627.2 ± 0.3 Da, calculated mass 5626.6 Da (average isotopes). The UV absorbance was monitored at 214 nm, and the m/z data was acquired over the marked region in the chromatogram. LC-MS analysis was carried out with Method A depicted in section 1.2.

5. Chemical synthesis of Caged Max

5.1 Chemical synthesis of Max-Nvoc via native chemical ligation (NCL)-desulfurization

5.1.1 NCL of segments 1 and 2

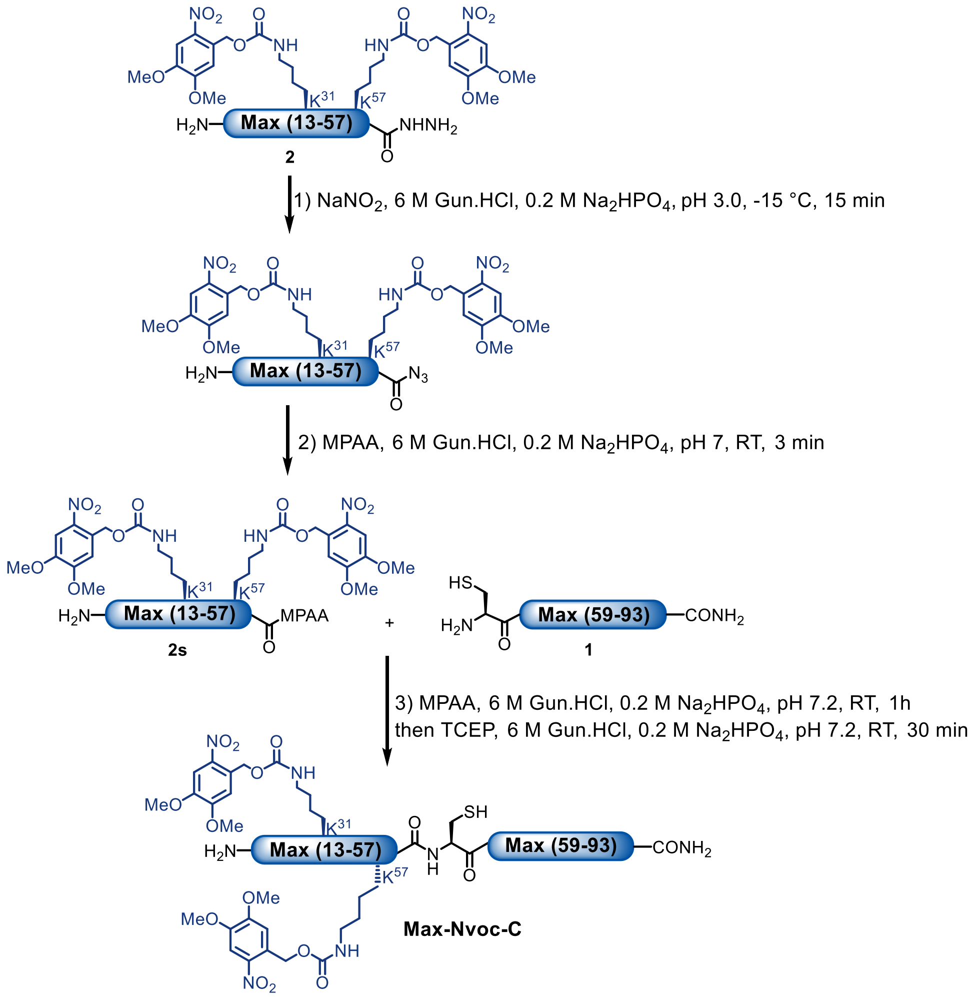

Segment **2** MaxK31NvocK57Nvoc(13-57)-$NHNH_2$ (3.3 mg, 1.3 equiv., 0.6 µmol, 7.8 mM) was dissolved in 6 M Gun.HCl, 0.2 M Na_2HPO_4 buffer (75 µL) at pH 3.0 and cooled to -15 °C by placing in an ice/salt bath. 5 µL of $NaNO_2$ (10 equiv., 6.0 µmol, 1.2 M; based on **2**) dissolved in water was added to the reaction mixture and allowed to react for 15 min at -15 °C with gentle mixing in repeated intervals. After 15 min, 75 µL of MPAA (50 equiv., 30 µmol, 0.4 M; based on **2**) in 6 M Gun.HCl, 0.2 M Na_2HPO_4 buffer at pH 7 was added to the mixture and gently mixed for two to three minutes. Then, segment **1** Cys-Max(59-93) (2 mg, 1.0 equiv., 0.5 µmol, 3 mM) was dissolved in the reaction mixture and the pH was adjusted to 7.2 using 5 *N* NaOH. The mixture was then incubated for 1 h at 25 °C and then 75 µL of TCEP (40 equiv., 24 µmol, 0.3 M; based on **2**) in 6 M Gun.HCl, 0.2 M Na_2HPO_4 buffer at pH 7.2 was added and continued incubating for

30 min at 25 °C. The reaction was monitored using LC-MS (Method A described in Section 1.2).[5,6] After 1.5 h ligation, the reaction mixture was purified using RP-HPLC (Method B described in Section 1.3) affording 2 mg (0.2 µmol) of the final product **Max-Nvoc-C** as a white powder (44% yield, based on the limiting segment **1**).

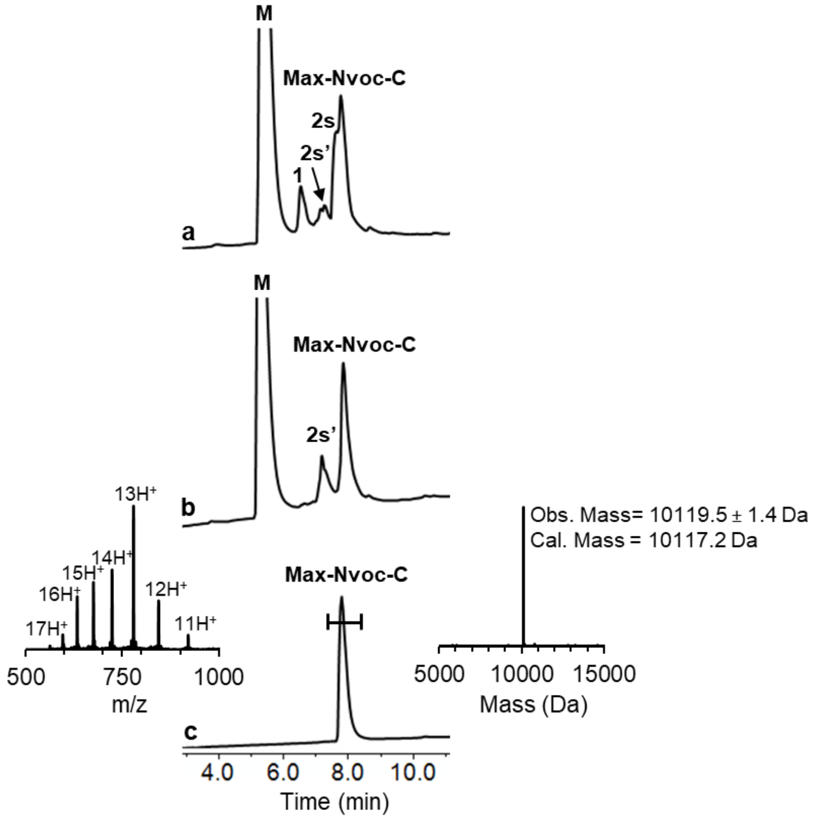

Figure S7. LC-MS analysis of the progress of the ligation of segment **1** and **2**. **(a)** ligation at t = 0 min; MaxK31NvocK57Nvoc(13-57) thioester segment (**2s**), hydrolysis of MaxK31NvocK57Nvoc(13-57) thioester (**2s'**), Cys-Max(59-93) segment (**1**), ligated product **Max-Nvoc-C** , and M=MPAA. **(b)** crude ligation reaction at t = 90 min. **(c)** RP-HPLC purified **Max-Nvoc-C** with the observed mass 10119.5 ± 1.4 Da, calculated mass 10117.2 Da (average isotopes). The UV absorbance was monitored at 214 nm, and the m/z data was acquired over the marked region in the chromatogram.

5.1.2 Desulfurization of Max-Nvoc-C

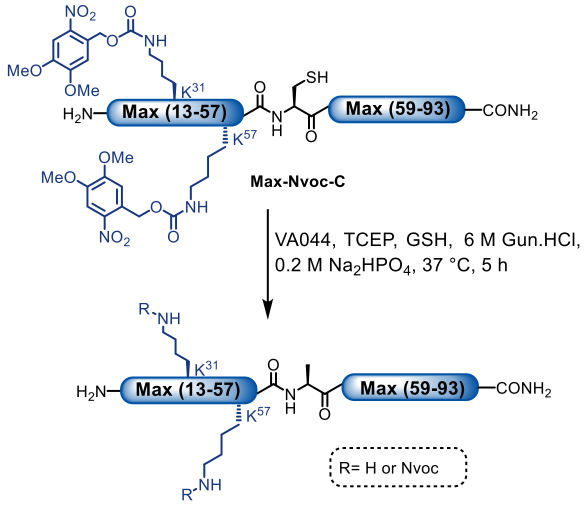

To a 1.5 mL Eppendorf tube was added **Max-Nvoc-C** (1 equiv., 5 µL, 4 mM), TCEP (125 equiv., 2 µL, 1.25 M), VA044 (100 equiv., 2 µL, 1 M), and L-Glutathione (GSH, 20 equiv., 1 µL, 400 mM). All added as a solution in 6 M Gun.HCl, 0.2 M Na_2HPO_4 buffer. The final concentrations of the major reaction components were the following: **Max-Nvoc-C** (2 mM); TCEP (250 mM); VA044 (200 mM); GSH (40 mM). The reaction was carried out at 37 °C.[7] The progress of the reaction was monitored by LC-MS using Method A (Section 1.2). Result was depicted in Figure S8.

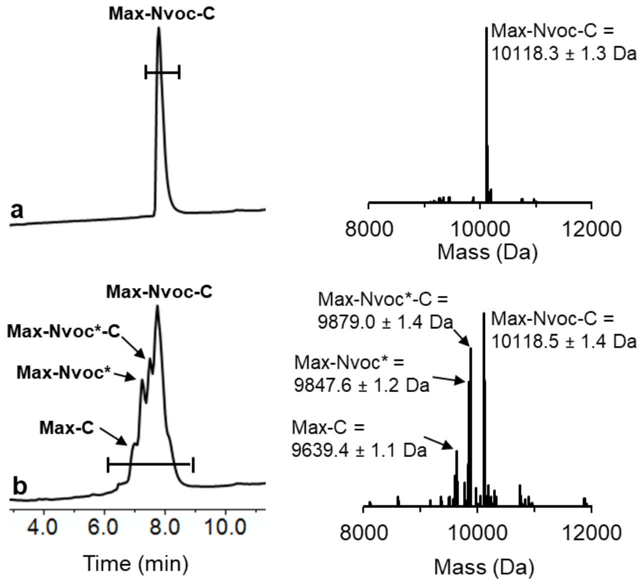

Figure S8. LC-MS analysis of desulfurization reaction of **Max-Nvoc-C**. **(a) Max-Nvoc-C** analog desulfurization at t = 0 min. **(b)** desulfurization at t = 5 hours; **Max-Nvoc*-C**: one Nvoc decomposed product; **Max-Nvoc*:** desulfurized **Max-Nvoc*-C**, and **Max-C**: both Nvoc decomposed product. The UV absorbance was monitored at 214 nm, and the deconvolution mass acquired over the marked region in the chromatogram.

Note: Multiple desulfurization attempts were conducted with different additives and conditions, including t-butyl mercaptan and alternative methods like low-energy visible light-induced desulfurization (LEnVLD), but none were successful.[8]

5.2 Chemical synthesis of Max-Nvoc via NCL and S-arylation

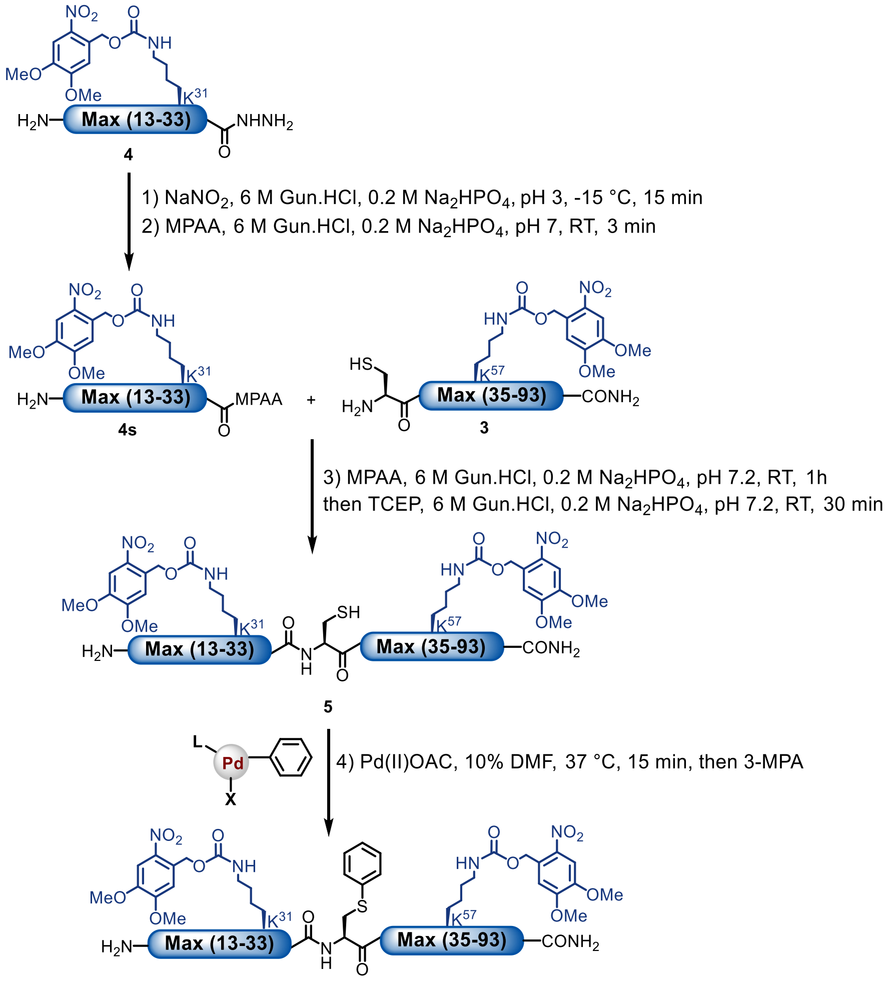

Segment **4** MaxK31Nvoc(13-33)-$NHNH_2$ (4 mg, 1.3 equiv., 1.4 µmol, 7.8 mM) was dissolved in 6 M Gun.HCl, 0.2 M Na_2HPO_4 buffer (183.5 µL) at pH 3.0 and cooled to -15 °C by placing in an ice/salt bath. 10 µL of $NaNO_2$ (10 equiv., 14.0 µmol, 1.4 M; based on **4**) dissolved in water was added to the reaction mixture and allowed to react for 15 min at -15 °C with gentle mixing in repeated intervals. After 15 min, 183.5 µL of MPAA (50 equiv., 70 µmol, 0.4 M; based on **4**) in 6 M Gun.HCl, 0.2 M Na_2HPO_4 buffer at pH 7 was added to the mixture and gently mixed for two to three minutes. Then, segment **3** Cys-MaxK57Nvoc(35-93) (8 mg, 1.0 equiv., 1.1 µmol, 3 mM) was dissolved in the reaction mixture and the pH was adjusted to 7.2 using 5 *N* NaOH. The mixture was then incubated for 1 h at 25 °C and then 183.5 µL of TCEP (40 equiv., 56 µmol, 0.3 M; based on **4**) in 6 M Gun.HCl, 0.2 M Na_2HPO_4 buffer at pH 7.2 was added and continued incubating for 30 min at 25 °C. The reaction was monitored using LC-MS (Method B described in Section 1.2). After 1.5 h ligation, the reaction mixture was desalted by pipetting the reaction mixture into a 10

kDa molecular weight cutoff spin filter (Amicon® Ultra- 2mL, 10K). The reaction mixture was diluted with a 6 M Gun.HCl, 0.2 M Na_2HPO_4 buffer (pH 7.2) to 2.0 mL and concentrated to 1 mL by Centrifuging the spin filter at 5000 rpm for 15 min. This process was repeated three times. After that, the reaction mixture was collected by reverse centrifuge. The reaction mixture was diluted to 250 µM, followed by addition of Pd(II)OAC (5 mg, 5 equiv., 5.5 µmol, 12.5 mM) which was dissolved in DMF (440 µL). The reaction was kept at 37 °C for 15 min. The reaction was monitored using LC-MS (Method B described in Section 1.2). Then, 3-MPA (5 equiv. compared with Pd(II)OAC complex) was added and kept for additional 10 min at 25 °C to quench reaction. Finally, the reaction mixture was purified by RP-HPLC (Method B as described in section 1.3) affording 3.2 mg (0.32 µmol) of the final product **Max-Nvoc** as a white powder (29% yield, based on the limiting segment **3**).

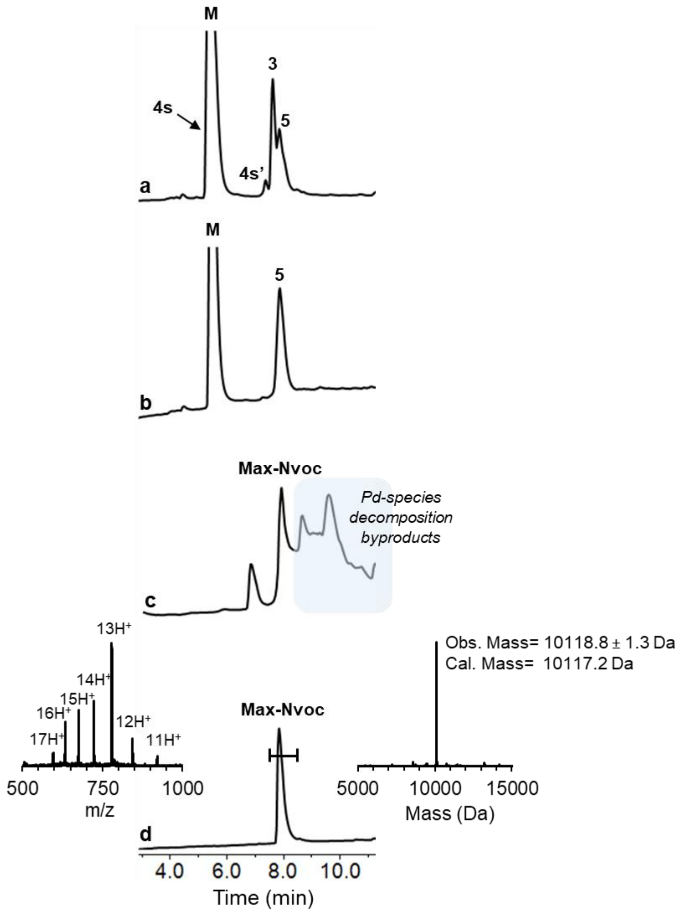

Figure S9. LC-MS analysis of the **Max-Nvoc** synthesis via NCL-S-arylation. **(a)** ligation at t = 0 min; MaxK31Nvoc(13-33) thioester segment (**4s**), hydrolysis of MaxK31Nvoc(13-33) thioester (**4s'**), Cys-MaxK57Nvoc(35-93) segment (**3**), ligated product MaxK31NvocK57Nvoc(13-93) (**5**), and M=MPAA. **(b)** Crude ligation reaction at t = 90 min. **(c)** Crude S-arylation reaction with Pd(II)OAC, at t = 15 min. **(d)** RP-HPLC purified **Max-Nvoc** with the observed mass 10118.8 ± 1.3 Da, calculated mass 10117.2 Da (average isotopes). The UV absorbance was monitored at 214 nm, and the m/z data was acquired over the marked region in the chromatogram.

6. Chemical synthesis of T-Myc and Native Max

6.1 Chemical synthesis of T-Myc via NCL-desulfurization

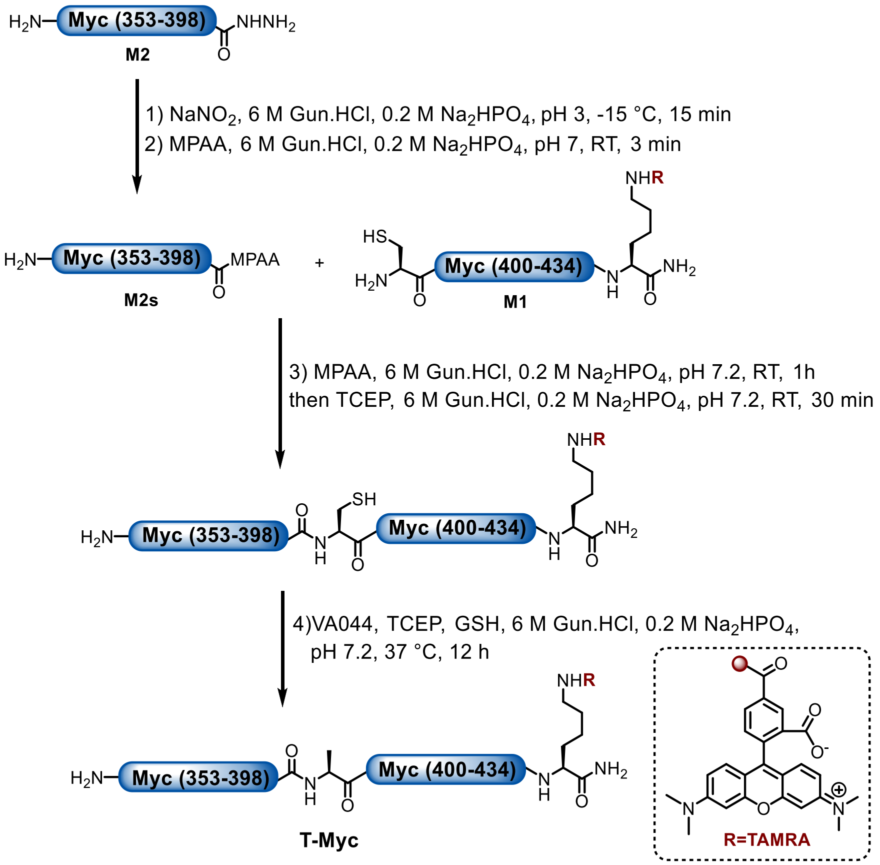

Segment **M2** Myc(353-398)-NHNH$_2$ (3.8 mg, 1.5 equiv., 0.68 µmol, 6.1 mM) was dissolved in 6 M Gun.HCl, 0.2 M Na_2HPO_4 buffer (112 µL) at pH 3.0 and cooled to -15 °C by placing in an ice/salt bath. 10 µL of $NaNO_2$ (10 equiv., 6.8 µmol, 0.68 M; based on **M2**) dissolved in water was added to the reaction mixture and allowed to react for 15 min at -15 °C with gentle mixing in repeated intervals. After 15 min, 112 µL of MPAA (50 equiv., 34 µmol, 0.3 M; based on **M2**) in 6 M Gun.HCl, 0.2 M Na_2HPO_4 buffer at pH 7 was added to the mixture and gently mixed for two to three minutes. Then, segment **M1** Cys-Myc(400-434)-T (2.2 mg, 1.0 equiv., 0.45 µmol, 2 mM) was dissolved in the reaction mixture and the pH was adjusted to 7.2 using 5 *N* NaOH. The mixture was then incubated for 1 h at 25 °C and then 112 µL of TCEP (40 equiv., 27 µmol, 0.24 M; based on **M2**) in 6 M Gun.HCl, 0.2 M Na_2HPO_4 buffer at pH 7.2 was added and continued incubating for 30 min at 25 °C. The reaction was monitored using LC-MS (Method B described in Section 1.2). After 1.5 h ligation, the reaction mixture was desalted by pipetting the reaction mixture into a 3 kDa molecular weight cutoff spin filter (Amicon® Ultra- 2mL, 3K). The reaction mixture was diluted with a 6 M Gun.HCl, 0.2 M Na_2HPO_4 buffer (pH 7.2) to 2.0 mL and concentrated to 0.5 mL by Centrifuging the spin

filter at 5000 rpm for 20 min. This process was repeated three times. After that, the reaction mixture was collected by reverse centrifuge and treated with VA044 (0.1 mmol, 200 mM), TCEP (0.13 mmol, 250 mM), and L-Glutathione (GSH, 30 µmol, 60 mM) for 12 h. The progress of the reaction was monitored by LC-MS using Method A (Section 1.2). After the completion of the reaction, purification was carried out using RP-HPLC (Method B described in Section 1.3) affording 1.3 mg (0.11 µmol) of the final product **T-Myc** as a pink powder (24% yield, based on the limiting segment **M1**).

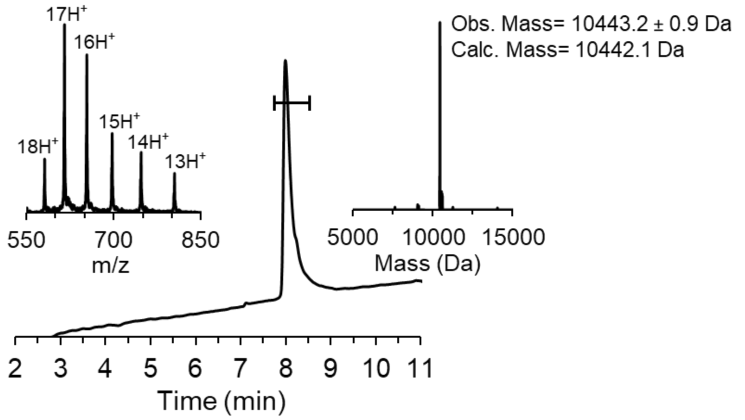

Figure S10. LC-MS analysis of segment **T-Myc** with the observed mass 10443.2 ± 0.9 Da, calculated mass 10442.1 Da (average isotopes). The UV absorbance was monitored at 214 nm, and the m/z data was acquired over the marked region in the chromatogram. LC-MS analysis was carried out with Method A depicted in section 1.2.

6.2 Chemical synthesis of native Max.

The synthesis was carried out as previously reported.[9] The LC-MS of the final product is shown below.

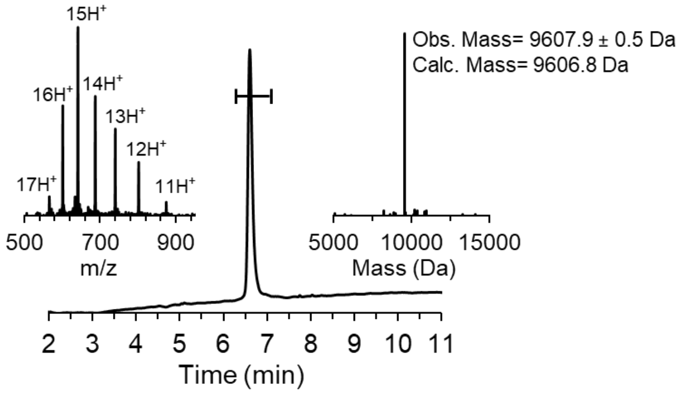

Figure S11. LC-MS analysis of segment **Native-Max** with the observed mass 9607.9 ± 0.5 Da, calculated mass 9606.8 Da (average isotopes). The UV absorbance was monitored at 214 nm, and the m/z data was acquired over the marked region in the chromatogram. LC-MS analysis was carried out with Method B depicted in section 1.2.

7. Decaging of Max-Nvoc analog

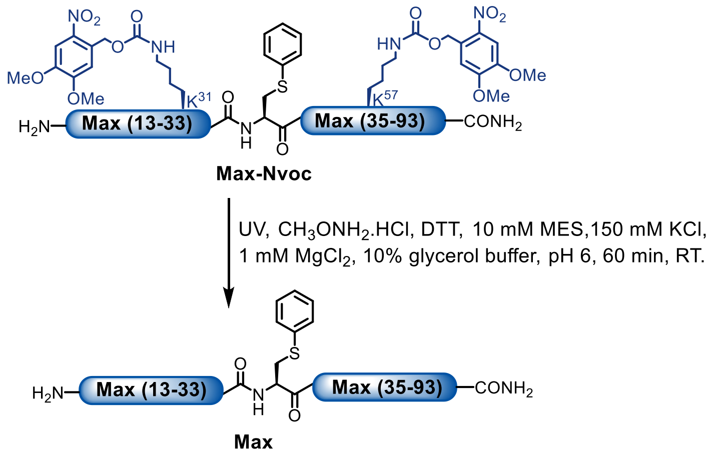

To remove the photolabile protecting groups, **Max-Nvoc** (1 equiv., 200 µM) was dissolved in 10 mM MES, 150 mM KCl, 1 mM $MgCl_2$, 10% glycerol buffer pH 6 or 1X PBS buffer pH 7, with or without additives (see table below). The mixture was irradiated with a UV lamp at 365 nm / 350 nm, for 1 h.[10] The reaction was monitored using LC-MS (Method B described in Section 1.2).

	Conditions	Yield*
1	UV 365 nm, MES buffer, 2 mM DTT, 40 mM CH_3ONH_2.HCl, pH 6, rt, 60 min	>95%
2	UV 365 nm, 1X PBS buffer, 2 mM DTT, 40 mM CH_3ONH_2.HCl, pH 7, rt, 60 min	99%
3	UV 350 nm, MES buffer, 2 mM DTT, 40 mM CH_3ONH_2.HCl pH 6, rt, 60 min	99%
4	UV 350 nm, MES buffer, pH 6, rt, 60 min	99%
5	UV 350 nm, MES buffer, pH 6, rt, 30 min	79%
6	UV 350 nm, 1X PBS buffer, pH 7, rt, 60 min	90%

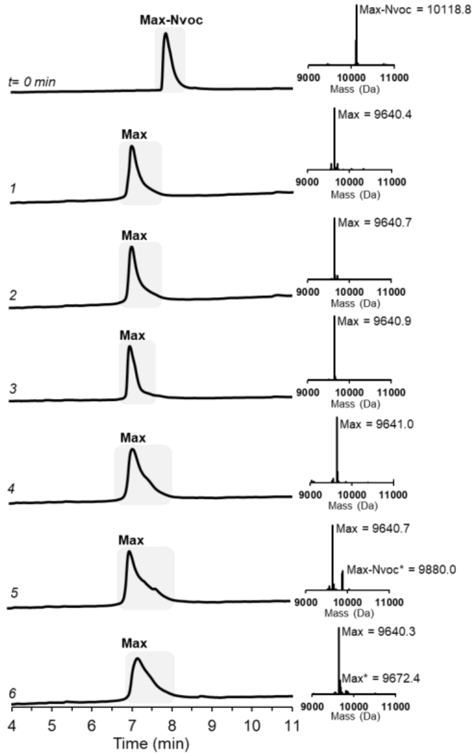

Figure S12. Table summarizing **Max-Nvoc** decaging conditions and conversion yields and corresponding LC analysis. *Conversion yields were determined by LC-MS analysis. Max-Nvoc*: corresponds to the removal of one Nvoc group. Max*: corresponds to oxidized Max. The UV absorbance was monitored at 214 nm, and the deconvolution mass was acquired over the marked region in the chromatogram (Method A depicted in section 1.2).

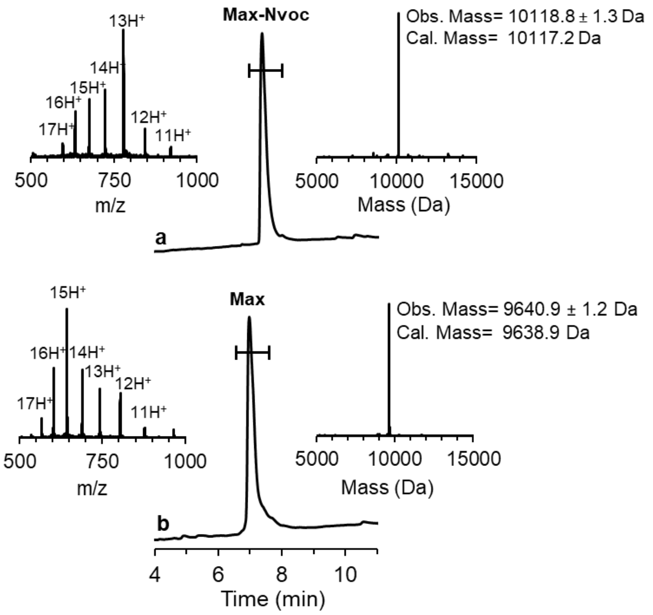

Figure S13. LC-MS analysis of Nvoc-decaging reaction of **Max-Nvoc** with optimized condition. **(a)** Nvoc decaging at t = 0 min. **(b)** Nvoc decaging at t = 60 min, Both Nvoc decaged product-**Max**. The UV absorbance was monitored at 214 nm, and the m/z data was acquired over the marked region in the chromatogram. LC-MS analysis was carried out with Method A depicted in section 1.2. Decaging conditions: UV 350 nm, MES buffer, 2 mM DTT, 40 mM CH_3ONH_2.HCl pH 6, rt.

8. DNA-Binding Analysis and Electrophoretic Mobility-Shift Assay (EMSA)

An E-box DNA probe (4.0 µL, 10.0 µM) was added to 0.6 mL Eppendorf tubes containing 36.0 µL, 34.0 µL, 33.0 µL, 32.0 µL, or 31.0 µL of 10 mM MES, 150 mM KCl, 1 mM $MgCl_2$, and 10% glycerol buffer (pH 6). The target protein analog, prepared in the same buffer, was then added at volumes of 2.0 µL, 3.0 µL, 4.0 µL, or 5.0 µL (40.0 µM) to tubes containing 34.0 µL, 32.0 µL, 30.0 µL, or 28.0 µL of buffer and 4 µL of E-box DNA probe, respectively. The final concentrations in the mixture were 1.0 µM for DNA and either 0 µM, 1.0 µM, 1.5 µM, 2.0 µM, or 2.5 µM for the protein dimer. Each tube was thoroughly mixed by pipetting up and down and then incubated at room temperature for 30 minutes or 45 minutes. Concurrently, a 10% TBE gel (1.0 mm thick, 10 wells) was prepared. After incubation, the DNA-binding activity of each protein analog was evaluated by EMSA. For this assay, 5.0 µL of the DNA-protein mixture was mixed with 1.0 µL of DNA Loading Dye (6X). 5.0 µL of this mixture was then loaded onto the 10% TBE polyacrylamide gel and electrophoresed at 90V for 40 minutes or 75 minutes. Following electrophoresis, the gel was washed three times with water (30 seconds each) to remove excess ions and visualized using an A2S Vilber Fusion FX imager for the fluorescent EMSA, followed by staining with Ethidium Bromide in 1x TBE buffer for 15 minutes at room temperature. Bands representing bound and unbound DNA were visualized using an A2S Vilber Fusion FX imager.

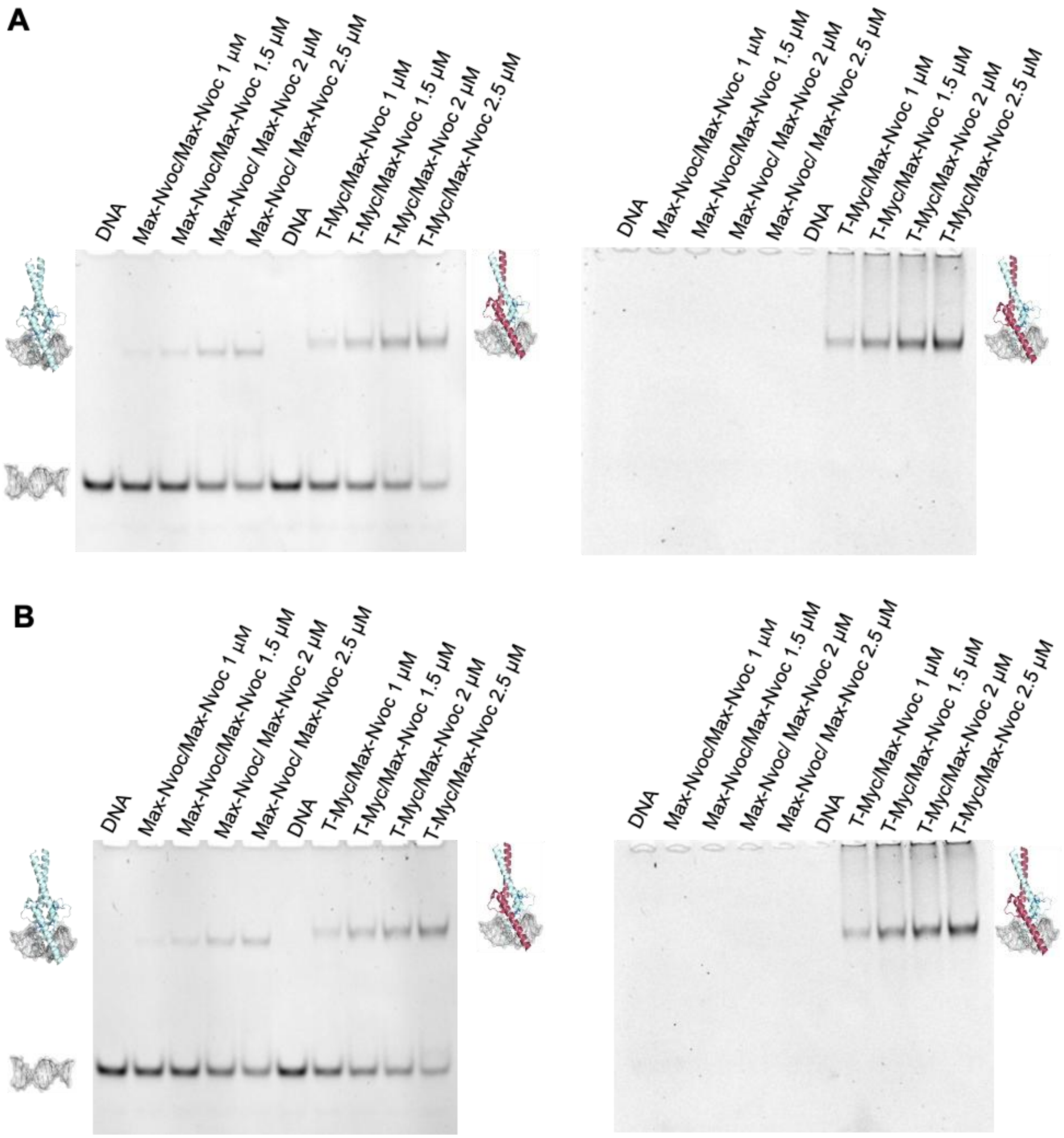

Figure S14. EMSA experiment of **Max-Nvoc** homodimer and heterodimer with **T-Myc**. **A**) EMSA experiment, DNA imaging via ethidium bromide (left) and T-Myc imaging via fluorescent group (right). **B**) Duplicate of the experiment. EMSA Conditions: 1 µM DNA probe and 0, 1, 1.5, 2, and 2.5 µM of homodimer of **Max-Nvoc** or **T-Myc/Max-Nvoc** heterodimer in 10 mM MES, 150 mM KCl, 1 mM $MgCl_2$, and 10% glycerol buffer (pH 6.0).

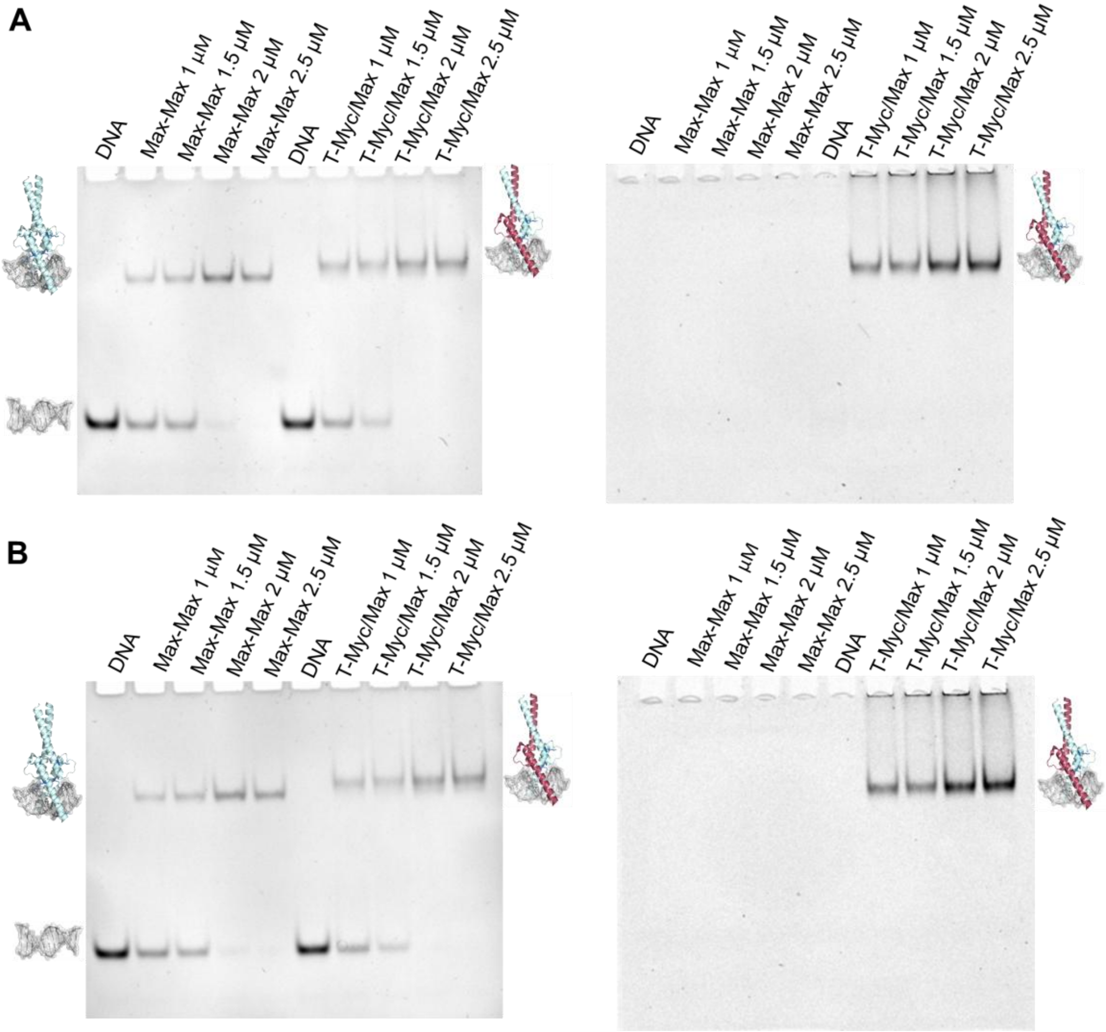

Figure S15. EMSA experiment of **Native-Max** homodimer and heterodimer with **T-Myc**. **A**) EMSA experiment, DNA imaging via ethidium bromide (left) and T-Myc imaging via fluorescent group (right). **B**) Duplicate of the experiment. EMSA Conditions: 1 µM DNA probe and 0, 1, 1.5, 2, and 2.5 µM of homodimer of **Native-Max** or **T-Myc/Native-Max** heterodimer in 10 mM MES, 150 mM KCl, 1 mM $MgCl_2$, and 10% glycerol buffer (pH 6.0).

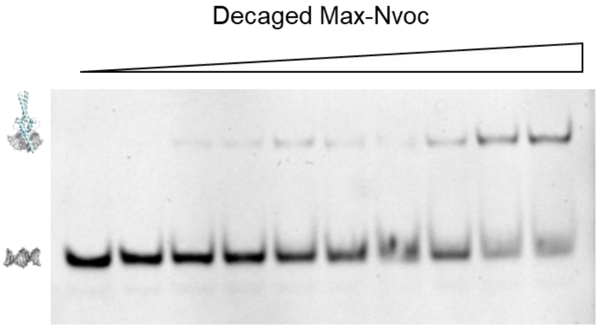

Figure S16. EMSA experiment of Decaged **Max-Nvoc** without any additives. Decaging conditions: UV 350 nm, MES buffer, pH 6, rt, 60 min. EMSA experiment conditions: 1 µM DNA probe and 0, 1, 1.5, 2, 2.5, 3, 3.5, 4, 4.5, and 5 µM of homodimer of decaged **Max-Nvoc** in 10 mM MES, 150 mM KCl, 1 mM $MgCl_2$, and 10% glycerol buffer (pH 6.0).

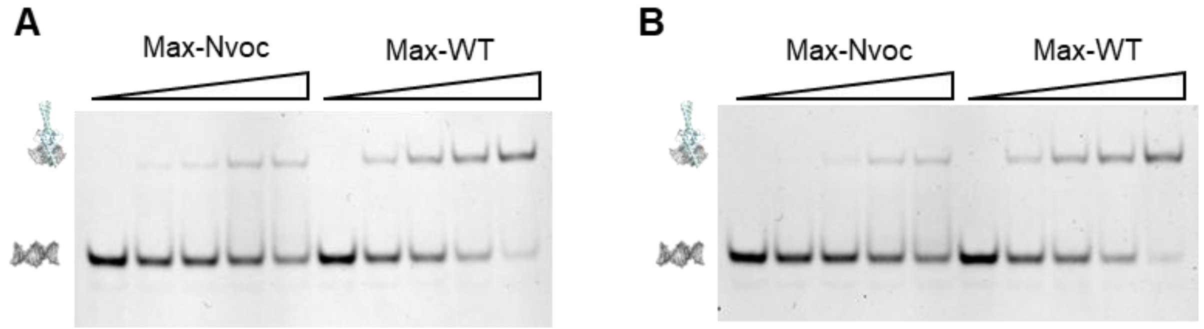

Figure S17. EMSA experiment of **Max-Nvoc** and **Max-WT**. **A**) EMSA experiment conditions: 1 µM DNA probe and 0, 1, 1.5, 2, and 2.5 µM of homodimer of Max-Nvoc or Max-WT in 10 mM MES, 150 mM KCl, 1 mM $MgCl_2$, and 10% glycerol buffer (pH 6.0). **B**) Duplicate of the experiment. Decaging conditions: UV 350 nm, MES buffer, 2 mM DTT, 40 mM CH_3ONH_2.HCl pH 6, rt, 60 min.

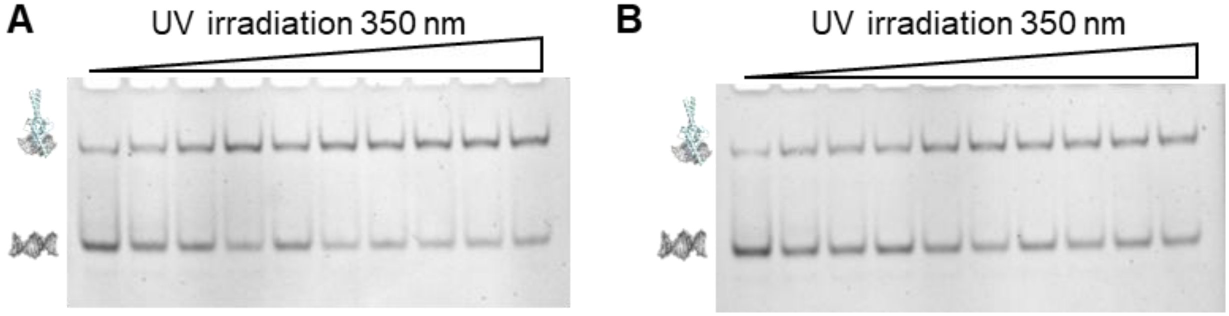

Figure S18. EMSA experiment of Max-Nvoc decaging in the presence of DNA. **A**) EMSA conditions: 1 µM DNA probe and 5 µM protein subjected to UV irradiation for varying time intervals ranging from 0 to 70 min in 10 mM MES, 150 mM KCl, 1 mM $MgCl_2$, and 10% glycerol buffer (pH 6.0). **B**) Duplicate of the experiment. The experiments were performed in independent replicates. Decaging conditions: UV 350 nm, MES buffer, 2 mM DTT, 40 mM CH_3ONH_2.HCl pH 6, rt.

9. Circular Dichroism (CD) Analysis

CD analysis was performed using a Chirascan circular dichroism spectrometer with a 0.1 mm path-length quartz cuvette. Max variants were prepared at a concentration of 5 µM dimer in a 50.0 µL of 10 mM MES, 150 mM KCl, 1 mM $MgCl_2$, and 10% glycerol buffer (pH 6.0). CD spectra of all samples were recorded in triplicates at 20 °C from 190 nm to 270 nm in a 1.0 nm step with 3.0 nm slit bandwidth and an averaging time of three seconds per wavelength.

For DNA binding studies, an annealed E-box DNA probe (10.0 µM) was prepared in the same buffer. A sample containing 5 µM DNA and protein dimer (1:1) in a final volume of 50.0 µL was then prepared. CD spectra of each complex were recorded under identical conditions.

10. Octet BioLayer Interferometry Binding Assay (BLI)

Biolayer interferometry (BLI) assays were performed using an Octet Red R4 System (ForteBio; Menlo Park,CA) in 96 well plates. Streptavidin Octet biosensors (ForteBio; Menlo Park, CA) were dipped into 0.1% BSA, 0.02% Tween-20, 1x PBS (kinetic buffer) for 10 min and then 60 more seconds in the kinetic buffer to obtain the baseline. Then the sensors were dipped into 200 µL 65 nM of biotinylated E-box DNA probe in the kinetic buffer for the loading step (300 sec). Sensors were then dipped into the kinetic buffer for 120 sec. Next, the tips were loaded with Max analog prepared in kinetic buffer at the indicated concentrations for 210 sec to obtain the association curve. Finally, the tips were dipped into the kinetic buffer for 480 sec to obtain the dissociation curve. Measurements were carried out at 25 °C. Data was analyzed within the ForteBio Data Analysis software. The association and dissociation curves are fitted with Fortebio Biosystems with a 1:1 binding model (global fitting algorism) to obtain the K_D. Kinetic K_D is reported. *Note: The decaged Max used for BLI analysis was taken directly from the crude reaction mixture without purification.*

Note: The decaging reaction and DNA-binding analysis of synthetic Max can be performed using either PBS buffer (pH 7) or MES buffer (pH 6).

11. References

(1) Lin, X.; Nithun, R. V.; Samanta, R.; Harel, O.; Jbara, M. Enabling Peptide Ligation at Aromatic Junction Mimics via Native Chemical Ligation and Palladium-Mediated S-Arylation. *Org Lett* **2023**, *25* (25), 4715–4719.

(2) Vinogradova, E. V.; Zhang, C.; Spokoyny, A. M.; Pentelute, B. L.; Buchwald, S. L. Organometallic Palladium Reagents for Cysteine Bioconjugation. *Nature* **2015**, *526* (7575), 687–691.

(3) Rojas, A. J.; Pentelute, B. L.; Buchwald, S. L. Water-Soluble Palladium Reagents for Cysteine *S* - Arylation under Ambient Aqueous Conditions. *Org Lett* **2017**, *19* (16), 4263–4266.

(4) Zheng, J.-S.; Tang, S.; Qi, Y.-K.; Wang, Z.-P.; Liu, L. Chemical Synthesis of Proteins Using Peptide Hydrazides as Thioester Surrogates. *Nat Protoc* **2013**, *8* (12), 2483–2495.

(5) Dawson, P. E.; Muir, T. W.; Clark-Lewis, I.; Kent, S. B. H. Synthesis of Proteins by Native Chemical Ligation. *Science (1979)* **1994**, *266* (5186), 776–779.

(6) Fang, G.; Li, Y.; Shen, F.; Huang, Y.; Li, J.; Lin, Y.; Cui, H.; Liu, L. Protein Chemical Synthesis by Ligation of Peptide Hydrazides. *Angewandte Chemie International Edition* **2011**, *50* (33), 7645–7649.

(7) Wan, Q.; Danishefsky, S. J. Free-Radical-Based, Specific Desulfurization of Cysteine: A Powerful Advance in the Synthesis of Polypeptides and Glycopolypeptides. *Angewandte Chemie International Edition* **2007**, *46* (48), 9248–9252.

(8) Han, D.; Deng, X.; Cui, Y.; Zhu, X.; Deng, G.; Liang, L.; Chu, G.; Liu, L. Superfast Protein Desulfurization Triggered by Low-Energy Visible Light. *Angewandte Chemie International Edition* **2025**, *64* (24).

(9) Harel, O.; Nadal-Bufi, F.; Nithun, R. V.; Minyi Yao, Y.; Afek, A.; Vendrell, M.; Jbara, M. Chemical Engineering of Transcription Factors Uncovered Cell-Permeable µMax Modulators. *Journal of the American Chemical Society* **2025**, 11 (13).

(10) Jbara, M.; Seenaiah, M.; Brik, A. Solid Phase Chemical Ligation Employing a Rink Amide Linker for the Synthesis of Histone H2B Protein. *Chem. Commun.* **2014**, *50* (83), 12534–12537.
